# Clinical Spectrum, Heteroplasmy‐Phenotype Correlation, and Prognosis of the *MT‐ND3* m.*10191 T* > C Mutation

**DOI:** 10.1002/cns.70997

**Published:** 2026-06-19

**Authors:** Zimeng He, Huafang Jiang, Tongyue Li, Yang Liu, Ying Zou, Chaolong Xu, Zhimei Liu, Danmin Shen, Ruoyu Duan, Minhan Song, Yunxi Zhang, Zixuan Zhang, Mingxi Sun, Rui Shen, Hong Xue, Fang Fang

**Affiliations:** ^1^ Department of Neurology, Beijing Children's Hospital Capital Medical University, National Center for Children's Health Beijing China; ^2^ Department of Pediatrics Weifang Maternal and Child Health Hospital Weifang Shandong China; ^3^ Department of Genetics Children's Hospital of Fudan University Shanghai China

**Keywords:** complex I deficiency, leigh syndrome, m.10191 T > C, mitochondrial encephalomyopathy with lactate acidosis and stroke‐like episodes (MELAS), *MT‐ND3*

## Abstract

**Aim:**

To systematically characterize the phenotypic spectrum, neuroimaging features, heteroplasmy‐phenotype correlation, and prognosis of the m.10191 T > C mutation.

**Methods:**

We collected and analyzed data from 52 patients (14 newly recruited; 38 from literature). Phenotypes were pre‐classified as Leigh syndrome (LS), Leigh‐like syndrome (LLS), MELAS/LS overlap syndrome, and MELAS‐like syndrome. Neuroimaging data were subjected to statistical analysis to explore inter‐lesional associations and lesion‐symptom correlations. Heteroplasmy level underwent k‐means clustering and latent class analysis (LCA) to define data‐driven subgroups and model genotype–phenotype correlations. Prognostic factors were evaluated through Bayesian logistic regression, and survival analysis was conducted.

**Results:**

The cohort exhibited phenotypic heterogeneity, dominated by LS (46.2%). Key features included epilepsy, developmental delay, and dystonia. Globus pallidus involvement frequently co‐occurred with midbrain and pontine lesions. Heteroplasmy level differed significantly across phenotypes. LCA identified three classes corresponding to clinical phenotypes. High heteroplasmy level, medullary involvement, and severe hyperlactatemia were associated with disease progression. Survival analysis indicated a 5 year survival rate of 80.0%, with high heteroplasmy level, hypotonia, and cerebellar lesions predicting poorer survival.

**Interpretation:**

The m.10191 T > C mutation is linked to a continuous clinical spectrum correlated with heteroplasmy level. Specific clinical and neuroimaging features serve as valuable biomarkers for phenotypic classification and prognostic assessment.

## Graphical abstract

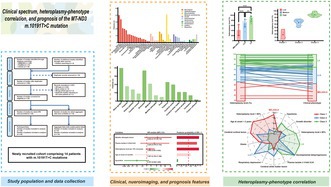

## 1 Introduction

Mitochondrial complex I (CI), the largest component of the respiratory chain, plays a vital role in the electron transfer from NADH to ubiquinone and drives proton translocation across the membrane [1]. CI is composed of seven mitochondrial DNA‐encoded subunits and at least 38 nuclear DNA‐encoded subunits, among which ND3 (encoded by *MT‐ND3*) is an essential mitochondrial‐encoded subunit. Isolated CI deficiency represents the most common genetic cause of oxidative phosphorylation (OXPHOS) disorders, and most manifest clinically as Leigh syndrome (LS) [2]. Notably, mutations in *MT‐ND3* have emerged as a frequent cause of CI deficiency, with several mutation sites identified, including m.10191 T > C, m.10197G > A, and m.10158 T > C [3, 4, 5]. The m.10191 T > C mutation substitutes a hydrophobic alanine with a hydrophilic threonine (Ser45Pro), disrupting the hydrophobic α‐helical domain of ND3. This disruption impairs its interactions with core CI subunits, leading to impaired enzyme catalytic activity and consequent CI dysfunction [5, 6]. This mutation was first reported in 2001 in an adult patient manifesting a MELAS‐like progressive mitochondrial disorder [7]. Subsequent studies have linked this mutation to a broad spectrum of mitochondrial diseases (MDs), ranging from typical LS to MELAS phenotypes [8].

The m.10191 T > C mutation is currently recognized as a pathogenic variant associated with LS, Leigh‐like syndrome (LLS), MELAS/LS overlap syndrome (MELAS/LS), and MELAS‐like syndrome [5, 8, 9]. However, despite its significance within the *MT‐ND3* gene, the complete clinical landscape of this mutation remains incompletely understood. Previously, the m.10191 T > C mutation has only been documented in isolated case reports and small case series, with the largest cohort to date comprising only six patients [10]. These studies primarily provided individual clinical descriptions; thus, systematic characterization of the complete phenotypic spectrum, heteroplasmy‐phenotype correlations, neuroimaging features, and prognostic indicators remains lacking. Specifically, it is unclear whether heteroplasmy levels correlate with disease phenotype, which neuroimaging lesion patterns are characteristic, and what factors predict prognosis. To address these gaps, we assembled the largest cohort to date (*n* = 52) by integrating 14 newly recruited patients with 38 systematically reviewed literature cases. Methodologically, we employed advanced analytical techniques, including k‐means clustering, latent class analysis (LCA), and multivariable Bayesian logistic regression. These approaches allowed us to investigate heteroplasmy‐phenotype associations, identify data‐driven patient subgroups, and evaluate independent prognostic factors through robust statistical validation. This study aims to provide a feasible research paradigm for rare mitochondrial DNA mutations by integrating literature‐derived and original data, applying diverse statistical methods, and establishing a comprehensive analytical framework that includes clinical phenotyping, neuroimaging characterization, genotype–phenotype correlation, and prognostic assessment. This paradigm not only enables a comprehensive understanding of the disease spectrum associated with the m.10191 T > C mutation, but also provides a valuable reference for research on other rare mitochondrial DNA mutations.

## 2 Methods

### 2.1 Study Population and Data Collection

**FIGURE 1:**
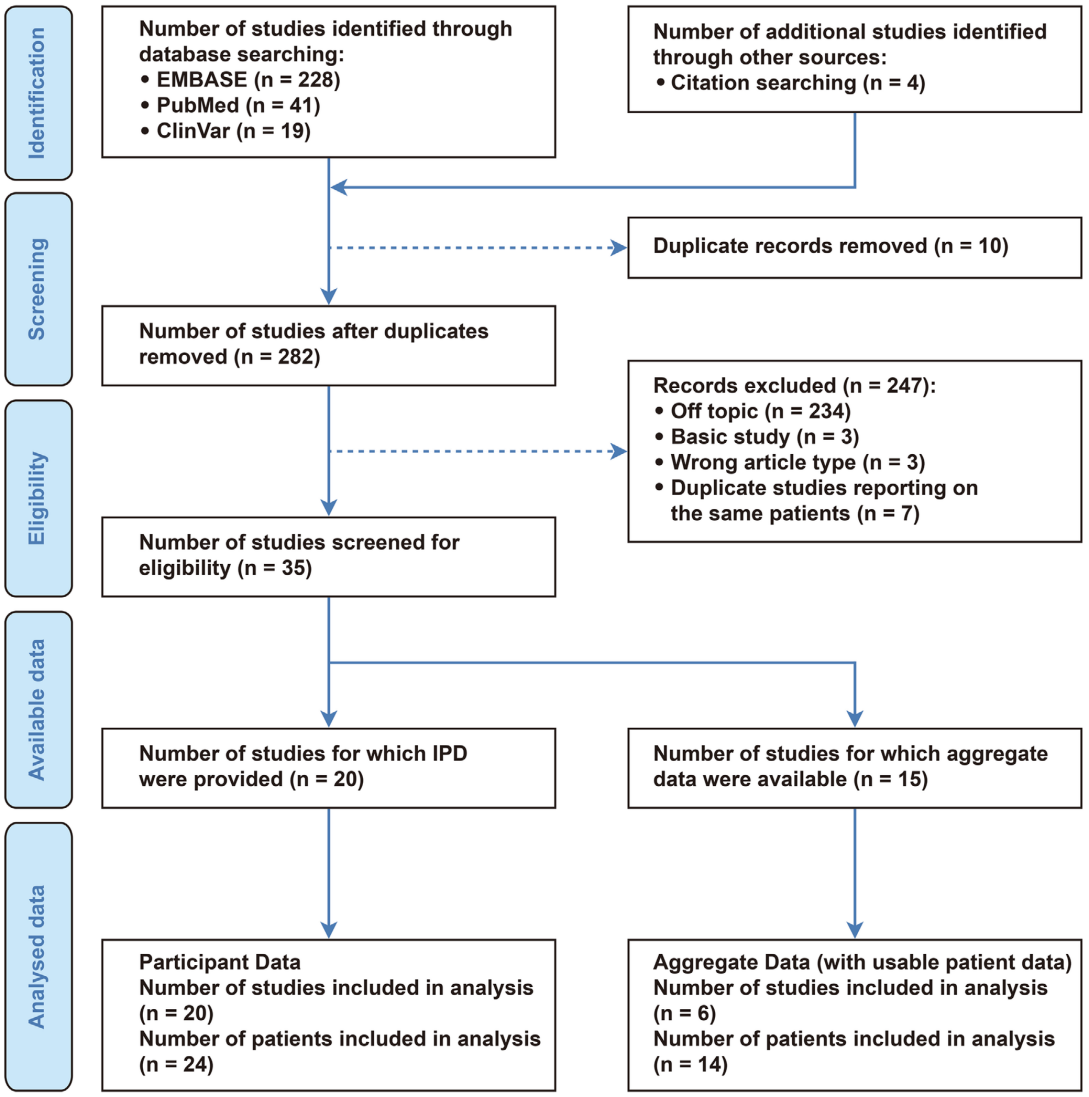
Flow diagram for literature review following the PRISMA‐IPD (Preferred Reporting Items for Systematic Reviews and Meta‐Analyses of individual participant data) guidelines [11].

The study population was derived from two sources. First, we assembled a newly recruited cohort comprising 14 patients with genetically confirmed m.10191 T > C mutations and MD phenotypes. Second, previously reported cases were identified through a systematic literature review, which included all published patients carrying the m.10191 T > C mutation (*n* = 38), irrespective of data completeness. The literature review flowchart is illustrated in Figure 1, and the complete methodology is detailed in Appendix S1. A standardized proforma was used to collect comprehensive data, encompassing demographic and clinical characteristics (sex, age at onset, initial presenting symptoms, clinical manifestations, family history, treatment, prognosis, and outcome); laboratory and metabolic findings (plasma and cerebrospinal fluid lactate levels, muscle histology, and tissue biochemistry); genetic features (heteroplasmy level and transmission pattern); and neuroimaging features. For cases identified from the literature, unavailable or indeterminable variables were marked as “unknown”.

### 2.2 Diagnostic Criteria and Phenotypic Classification

Based on clinical and imaging features, patients were categorized into one of four phenotypic groups using the following criteria:

1. LS: Satisfied all three diagnostic criteria for LS [12]: (a) neurodegenerative symptoms (e.g., developmental delay/regression); (b) biochemical abnormalities (elevated lactate and/or mitochondrial energy metabolism defect); and (c) characteristic MRI findings (bilateral basal ganglia and/or brainstem lesions).
2. LLS: Met two of the three LS diagnostic criteria [13]. Unmet criteria included the absence of developmental delay/regression, normal lactate levels/CI activity, or atypical MRI features (cerebral cortical or white matter lesions). Cases with cortical lesions were only included if they had no history of clinical stroke‐like episodes (SLEs).
3. MELAS/LS: Presented with clinical SLEs and corresponding cortical lesions on MRI, while also exhibiting the characteristic bilateral basal ganglia and/or brainstem lesions of LS [14].
4. MELAS‐like: Exhibited clinical SLEs and corresponding cortical lesions on MRI, but lacked the characteristic bilateral basal ganglia and/or brainstem lesions of LS.

For literature‐derived cases, the original diagnostic designation was retained when essential information required for reclassification (e.g., lactate levels) was unavailable. Additionally, one previously reported patient who could not be classified was defined as having nonspecific MD [15].

### 2.3 Analysis of Heteroplasmy Level‐Phenotype Correlation

Only patients with available heteroplasmy level data were included in the analysis. Heteroplasmy level of the m.10191 T > C mutation was primarily quantified in blood samples; for a small subset of literature‐sourced patients lacking blood data (*n* = 6), heteroplasmy levels measured in other tissues (skeletal muscle or fibroblasts) were used. First, patients were stratified according to predefined clinical phenotypes into LS, LLS, and MELAS/LS groups (for statistical purposes, the single MELAS‐like patient with available heteroplasmy level data was merged into the MELAS/LS group). Group differences in heteroplasmy level were then compared. Second, k‐means clustering (k = 3) was conducted on heteroplasmy level data, obtaining three clusters corresponding to high, medium, and low heteroplasmy level groups. The classification consistency between heteroplasmy level groupings (data‐driven) and phenotypic groupings (clinically predefined) was further compared to validate heteroplasmy level‐phenotype correlations. Results were visualized through parallel coordinate plots and quantified using cluster purity and correct classification rate. Finally, to explore potential associations among heteroplasmy level, clinical phenotypes, and specific clinical manifestations, we employed LCA. Guided by the heteroplasmy level distribution observed in our cluster analysis and prior knowledge of mitochondrial heteroplasmy thresholds [16, 17], the heteroplasmy level data were converted into a three‐category variable: low (< 60%), medium (60%–80%), and high (≥ 80%). Clinical phenotype (LS, LLS, or MELAS/LS) was similarly treated as a three‐category variable. Prior to LCA model construction, clinical manifestations were prescreened (see Results), and only variables with the highest discriminatory value for phenotypic differentiation were retained (Given that cortical lesions served as a key marker for defining phenotypic subgroups in this study, patients were first divided into cortical‐involved and non‐involved groups for comparison. Variables with a missing rate < 20% and an uncorrected *p* value < 0.05 that showed between‐group differences were selected for subsequent LCA). These included nine binary variables: age at onset > 2 years, developmental delay/regression, hypotonia, ataxia, growth disorder, respiratory depression, plasma lactate ≥ 3‐fold the upper limit of normal (ULN), cerebral cortical lesions, and cerebral white matter lesions.

### 2.4 Disease Course and Prognostic Analysis

Disease course was categorized into three categories: improvement, stability, or progression. For patients in the newly recruited cohort, disease course was assessed at regular follow‐ups based on parental reports of developmental milestone gain/loss and changes in clinical symptoms. For literature‐derived cases, disease course was determined through an integrated review of reported clinical evolution and imaging lesion changes. Cases with insufficient data for such assessment were marked as “unknown”. We further employed multivariable Bayesian logistic regression to explore the associations between disease progression and clinical, metabolic, neuroimaging, and genetic factors. Clinical outcome was defined as death or survival. Survival status was ascertained at the last follow‐up or extracted from published reports when explicitly documented; otherwise, it was recorded as “unknown”. Subsequently, survival analysis was conducted using all available time‐to‐event data from the entire cohort, with additional subgroup analyses performed.

### 2.5 Statistical Analysis

For all statistical analyses, variables with missing data marked as “unknown” were excluded, and only clinical, metabolic, and neuroimaging features reported in ≥ 5 patients were included for analysis. Data normality was assessed using the Shapiro–Wilk test. Normally distributed continuous data are expressed as mean ± standard deviation (SD) and analyzed by independent samples *t*‐tests; non‐normally distributed continuous data are presented as median (interquartile range [IQR]) and analyzed using the non‐parametric Mann–Whitney U test. Categorical data are expressed as counts and percentages, and compared using the chi‐square (χ^2^) or Fisher's exact test, as appropriate. Univariable logistic regression was applied to analyze associations between lesions in specific brain structures on MRI (brainstem, basal ganglia, medulla, cerebellum, and cerebral cortex) and their corresponding clinical symptoms. In LCA, missing data were handled using full information maximum likelihood estimator. Model selection was performed by sequential comparison of models with one to four latent classes. The optimal number of classes was determined using the Lo–Mendell–Rubin likelihood ratio test (LMR‐LRT), bootstrapped likelihood ratio test (BLRT), Akaike information criterion (AIC), Bayesian information criterion (BIC), entropy, and clinical interpretability. Furthermore, to assess the robustness of the LCA solution, we performed a two‐step cluster analysis using the same indicators. The two‐step cluster algorithm applies listwise deletion for missing data; therefore, only patients with complete data on all indicators were included. Multivariable Bayesian logistic regression was restricted to cases with complete information. Weakly informative normal priors with a Normal (0, 2.5^2^) distribution were assigned to regression coefficients, and posterior distributions were estimated via Markov chain Monte Carlo (MCMC) sampling (4 chains, 4000 iterations per chain, 1000 warm‐up draws) [18]. Results are reported as posterior odds ratios (ORs) with 95% credible intervals (CrI); evidence strength was classified by the posterior probability *P* (OR > 1) as strong (≥ 0.9), moderate (0.8–0.9), or weak/inconclusive (< 0.8). Survival analysis was conducted using the Kaplan–Meier method, and differences between subgroups were compared using the log‐rank test. All tests were two‐tailed, with *p* < 0.05 considered statistically significant. To address multiple comparisons, *p* values were adjusted using either the Bonferroni correction or the Benjamini‐Hochberg false discovery rate (FDR) procedure, as appropriate. LCA was performed using Mplus (version 8.3); all other statistical analyses and visualizations were performed using IBM SPSS Statistics (version 27.0.1), R (version 4.4.2), and GraphPad Prism (version 10.1.2).

### 2.6 Sensitivity Analysis

We conducted two sets of post hoc sensitivity analyses to verify the robustness of our findings. First, to eliminate potential bias introduced by literature‐derived cases, the analyses were restricted to the newly recruited patient cohort. Within this subset, core analyses (pairwise lesion association, heteroplasmy–phenotype correlation, and prognosis analysis) were repeated. Due to the limited sample size, LCA and Bayesian logistic regression were not performed; instead, only univariate analyses of prognostic factors were conducted. Second, to address tissue‐specific variability, we repeated the core analyses using exclusively blood‐derived heteroplasmy data, excluding six patients whose heteroplasmy was measured in muscle or fibroblasts. All sensitivity analyses adhered strictly to the statistical procedures described in the primary analysis.

## 3 Results

### 3.1 Study Population

A total of 52 patients carrying the m.10191 T > C mutation were included in this study, comprising 14 newly recruited cases and 38 previously reported cases [5, 7, 8, 9, 10, 15, 19, 20, 21, 22, 23, 24, 25, 26, 27, 28, 29, 30, 31, 32, 33, 34, 35, 36, 37, 38]. After excluding cases with missing general demographic information, the cohort included 28 males (56.0%) and 22 females (44.0%). Seven patients reported a positive family history (relatives exhibited symptoms potentially related to mitochondrial dysfunction, such as hearing loss, epilepsy, or diabetes). The mutation was confirmed as de novo in 20 patients (58.8%) and maternally inherited in 14 patients (41.2%). Individual‐level data for all patients are detailed in Table 1 (new cohort) and Table S1 (literature cohort).

**TABLE 1:** Characteristics of the 14 newly collected patients with the m.10191 T > C mutation.

Characteristics	1	2	3	4	5	6	7	8	9	10	11	12	13	14
Phenotype spectrum	LS	LS	LLS	LLS	LLS	LS	LS	LLS	MELAS/LS	LLS	LLS	LS	LLS	LLS
Sex	Male	Male	Female	Male	Female	Male	Male	Male	Male	Female	Male	Male	Male	Male
Transmission	De novo	De novo	De novo	Maternal	Maternal	De novo	Maternal	De novo	De novo	De novo	De novo	De novo	Maternal	Maternal
Heteroplasmy level	88.0%	89.3%	76.0%	70.8%	73.9%	88.6%	73.0%	49.5%	44.7%	71.3%	83.5%	93.7%	56.4%	86.9%
Age at onset	4 m	1y	5 m	6y	1y10m	5 m	1y6m	1y6m	3y8m	3y	4 m	2y7m	2y	11 m
Family history	—	—	+	—	—	—	—	—	—	—	—	—	—	—
Dev delay	+	+	+	—	+	+	+	+	—	—	+	+	+	+
Dev regression	—	+	—	—	+	+	—	+	—	—	—	+	+	—
Cognitive impairment	+	NA	+	—	+	+	+	+	—	—	+	+	+	—
Seizure	+	+	+	—	—	+	—	+	+	—	+	+	+	—
Stroke‐like episode	—	—	—	—	—	—	—	—	+	—	—	—	—	—
Gait disturbance	—	—	—	+	—	+	+	+	+	—	—	+	—	—
Dystonia	+	+	—	+	+	—	—	+	—	+	—	—	+	+
Hypotonia	—	+	—	—	—	+	—	—	—	+	—	—	—	—
Ataxia	—	—	—	+	—	—	—	—	—	—	—	—	+	—
Abnormal reflex	—	—	—	—	+	+	—	+	—	+	—	—	+	—
Dysarthria	—	—	—	+	+	—	+	+	—	+	+	—	—	—
Dysphagia	—	—	+	—	—	—	—	—	—	—	+	—	—	—
Muscle weakness	—	+	—	—	—	—	—	—	—	+	—	—	+	—
Involuntary movement	—	—	—	—	—	—	—	—	+	+	—	—	+	—
Nystagmus	+	—	—	—	—	+	+	+	—	—	+	—	—	—
Ophthalmoplegia	—	+	—	—	+	+	+	+	—	—	+	—	—	+
Ptosis	—	+	—	—	—	—	—	+	—	—	—	—	—	—
Hearing loss	—	—	—	—	—	—	—	—	—	—	+	—	—	—
Respiratory depression	+	+	—	—	—	—	—	—	—	—	+	—	+	—
Failure to thrive	+	+	+	+	—	—	—	—	—	+	—	—	—	—
Short stature	+	—	+	+	—	—	—	—	—	+	—	—	—	—
Gastrointestinal	+	—	+	—	+	—	—	—	—	+	—	—	—	+
Hematological	+	—	+	—	—	—	—	—	—	—	+	—	+	—
Cardiovascular	—	—	—	—	—	—	+	—	—	—	—	—	—	—
Genitourinary	—	—	—	—	—	—	+	—	—	—	—	—	—	—
Endocrine	—	—	—	—	—	—	—	—	—	—	+	—	—	—
Elevated plasma lactate	+	+	+	+		+	+	+	+	+	+	+	+	+
Elevated CSF lactate	ND	ND	ND	ND	ND	ND	ND	—	—	ND	+	+	ND	ND
Brain MRI														
Basal ganglia	—	+	—	+	+	+	+	+	—	+	—	+	+	+
Brainstem	+	+	+	+	+	+	+	+	+	+	+	+	+	+
Medulla oblongata	—	+	+	+	—	—	—	—	—	—	—	—	+	—
Thalamus	+	—	+	—	—	+	—	+	+	—	+	—	+	+
Cerebellum	—	+	—	—	—	—	—	—	—	—	—	—	+	—
Cerebral atrophy	—	—	+	—	—	—	—	—	—	—	—	—	—	—
Cerebral cortex	—	—	—	—	—	—	—	+	+	—	—	—	+	+
Cerebral white matter	—	—	+	—	—	—	—	+	—	—	+	—	—	—
Brain MRS	ND	ND	ND	ND	ND	Cho↑	ND	—	Lac, Cho↑	ND	—	Lac, Cho↑	ND	ND
Treatment	Cocktail	Cocktail	Cocktail	Cocktail	Cocktail	Cocktail	Cocktail	Cocktail	Cocktail	Cocktail	Cocktail	Cocktail	Cocktail	Cocktail
Disease course	Prog.	Prog.	Prog.	Prog.	Stable	Prog.	Improved	Improved	Improved	Stable	Prog.	Prog.	Prog.	Prog.
Outcome (age at last follow‐up/death)	Alive (11 y 4 m)	Dead (1 y 3 m)	Alive (8 y 1 m)	Alive (16 y 4 m)	Alive (6 y 10 m)	Dead (2 y 1 m)	Alive (5 y 5 m)	Alive (10 y 2 m)	Alive (8 y 11 m)	Alive (3 y 10 m)	Alive (1 y 7 m)	Alive (3 y 4 m)	Alive (9 y 7 m)	Alive (2 y 5 m)

Abbreviations: −, negative/normal finding; +, positive/abnormal finding; ↑, increased; Cho, Choline peak; CSF, cerebrospinal fluid; Dev, developmental; Lac, lactate peak; LLS, Leigh‐like syndrome; LS, Leigh syndrome; m, months; MELAS, mitochondrial encephalomyopathy with lactate acidosis and stroke‐like episodes; MELAS/LS, MELAS/LS overlap syndrome; MRI, magnetic resonance imaging; MRS, magnetic resonance spectroscopy; NA, not applicable; ND, not detected; Prog., progressive; y, years.

### 3.2 Clinical Features

**FIGURE 2:**
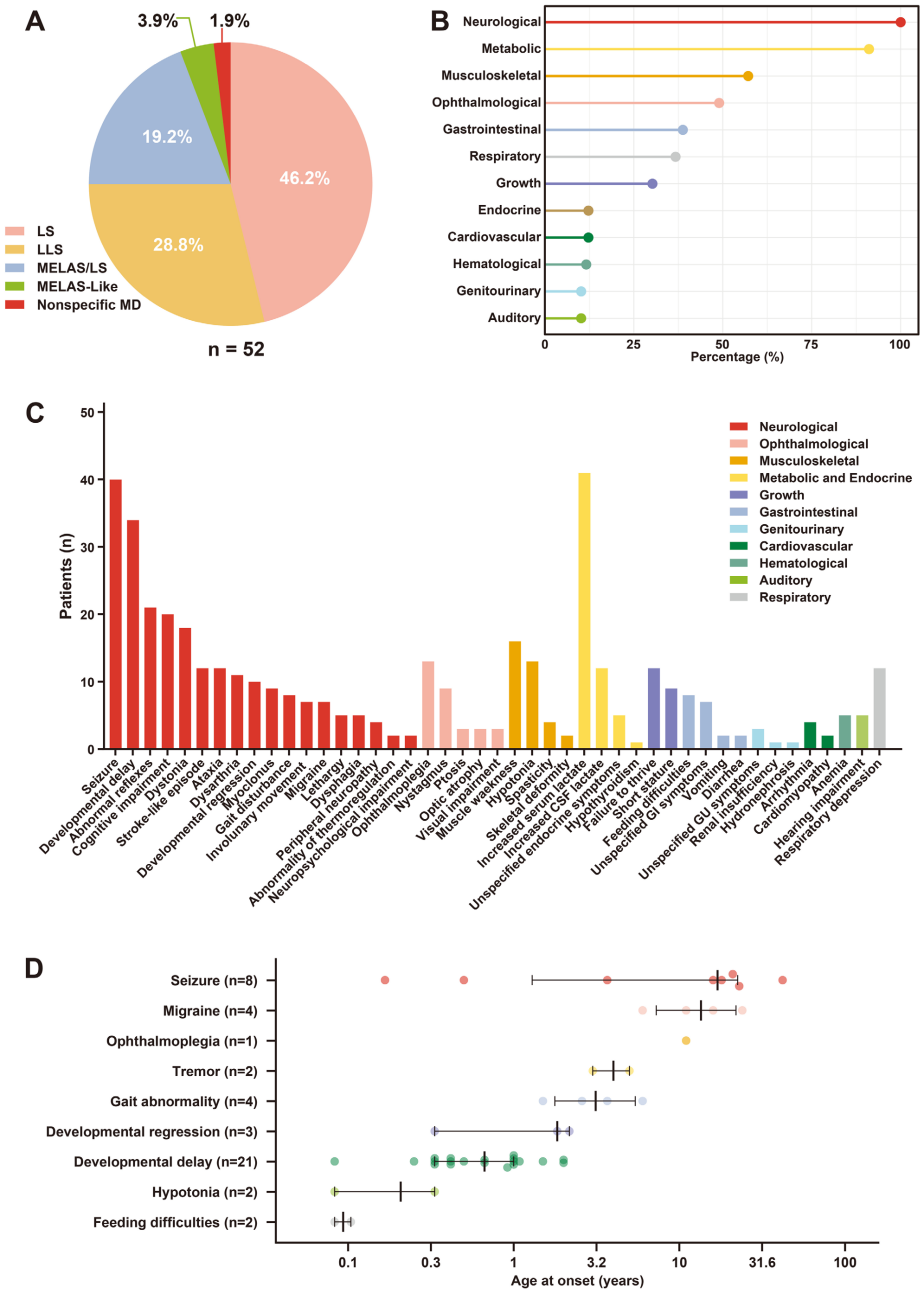
Clinical features of patients with the m.10191 T > C mutation. (A) Proportions of clinical phenotypes among all patients. (B) Frequency of involvement across different organ systems. (C) Spectrum of clinical manifestations by organ system. (D) Distribution of age at onset by initial symptoms. Age at onset is presented as median with 25th and 75th percentiles. The x‐axis is on a base‐10 logarithmic scale. LLS, Leigh‐like syndrome; LS, Leigh syndrome; MD, mitochondrial disease; MELAS, mitochondrial encephalomyopathy with lactate acidosis and stroke‐like episodes; MELAS/LS, MELAS/LS overlap syndrome.

Among all patients (*n* = 52), LS was the most frequent phenotype (46.2%), followed by LLS (28.8%), MELAS/LS (19.2%), MELAS‐like (3.9%), and nonspecific MD (1.9%) (Figure 2A). The neurological, metabolic, musculoskeletal, ophthalmological, and gastrointestinal systems were most commonly affected (Figure 2B). Among 49 evaluable patients, 38 (77.6%) developed multisystem involvement (≥ 3 systems) during their disease course. Clinical manifestations by organ system are summarized, with the most frequent features being increased serum lactate (41/45, 91.1%), epilepsy (40/49, 81.6%), developmental delay (34/49, 69.4%), cognitive impairment (20/34, 58.8%), and dystonia (18/43, 41.9%) (Figure 2C). Median age at onset among 50 patients with available data was 1.04 years (IQR 4.85; range 0–42). Onset occurred before 2 years of age in 31 patients (62.0%), presenting as LS (*n* = 20), LLS (*n* = 10), or MELAS/LS (*n* = 1), whereas 6 patients (12.0%) had adult‐onset disease exclusively with non‐LS phenotypes. Developmental delay was the most common initial symptom (21/47, 44.7%), with a median onset at 0.67 years (IQR 0.67), followed by epilepsy (8/47, 17.0%) at a median age of 17 years (IQR 21.21). The relationship between age at onset and the initial presenting symptoms is shown in Figure 2D.

### 3.3 Neuroimaging Features

**FIGURE 3:**
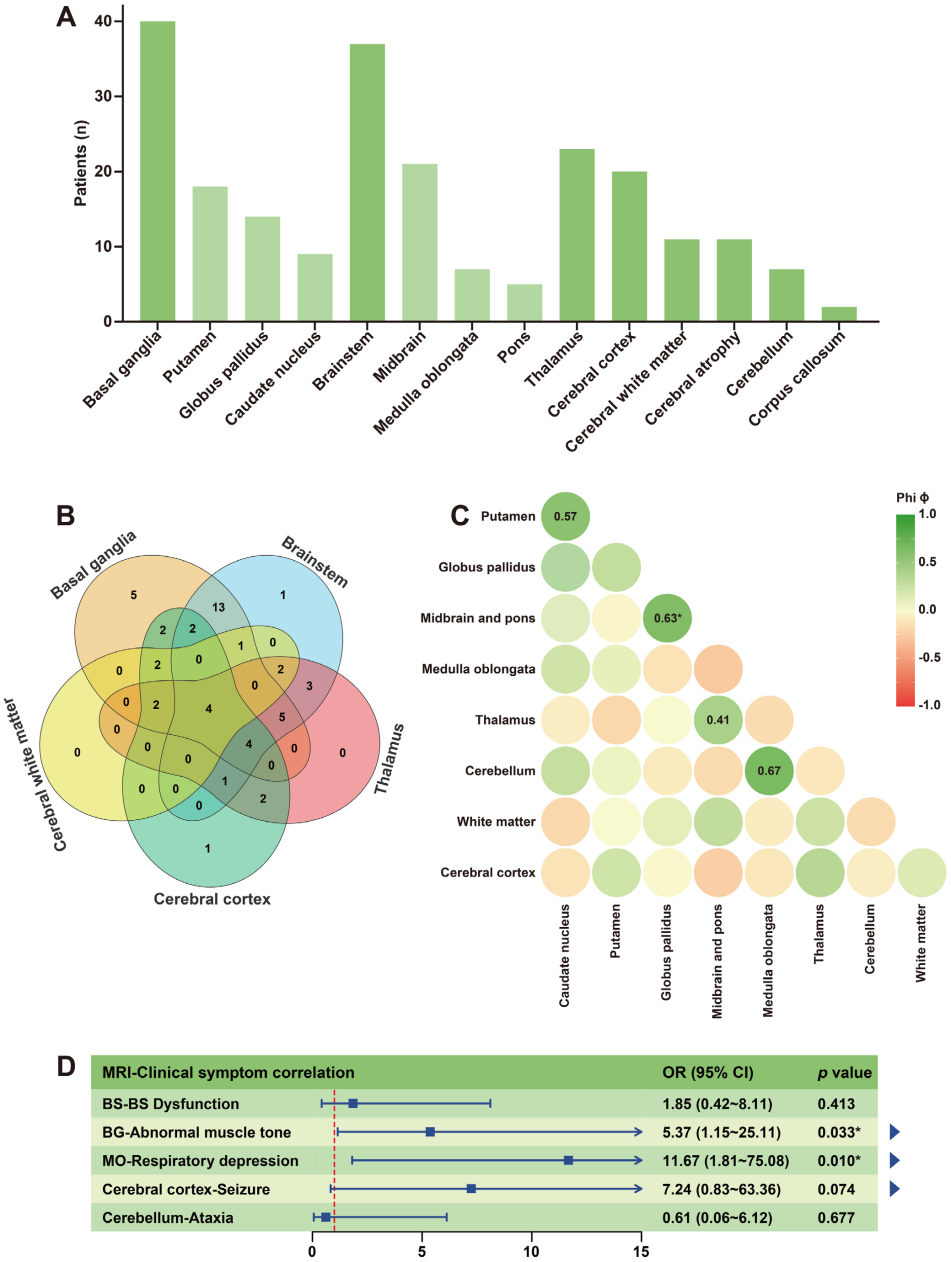
Neuroimaging features of patients with the m.10191 T > C mutation. (A) Location of MRI lesions across key brain structures. (B) Overlap of lesions among five major brain structures. (C) Pairwise association between specific lesion locations. Associations were quantified using the Phi coefficient and visualized in a correlation matrix. Green represents a positive association. Asterisks indicate statistically significant correlations (False discovery rate correction, *p < 0.05). (D) Association between specific brain lesions and clinical symptoms. Brainstem dysfunction encompassed respiratory depression, nystagmus, ophthalmoplegia, dysphagia, and dysarthria. Asterisks indicate statistically significant correlations (*p < 0.05). BG, basal ganglia; BS, brainstem; CI, confidence interval; MO, medulla oblongata; MRI, magnetic resonance imaging; OR, odds ratio.

Neuroimaging data were available for 50 patients. The most frequently involved sites on MRI were the basal ganglia, brainstem, thalamus, cerebral cortex, and cerebral white matter. Additionally, medullary involvement was present in seven patients, and cerebellar abnormalities (signal changes or atrophy) were observed in seven patients (Figure 3A). Combined basal ganglia and brainstem involvement was present in 29 patients, whereas lesions confined to the basal ganglia alone were found in five patients. Thalamic lesions commonly co‐occurred with basal ganglia and/or brainstem abnormalities, and white matter abnormalities frequently coexisted with cortical lesions. Four patients exhibited lesions across all five regions (basal ganglia, brainstem, thalamus, cerebral cortex, and white matter) (Figure 3B). Pairwise association analysis of lesions was performed in 30 patients with precisely defined lesions, excluding literature cases with only non‐specific reports of basal ganglia or brainstem involvement. The analysis demonstrated that globus pallidus involvement frequently co‐occurred with lesions in the midbrain and pons (Phi = 0.63, FDR‐corrected *p* = 0.03). An association was also observed between medullary and cerebellar lesions, although not statistically significant (Phi = 0.67, FDR‐corrected *p* = 0.06) (Figure 3C). We further assessed lesion‐clinical symptom associations. Significant correlations were identified for basal ganglia lesions with abnormal muscle tone (*p* = 0.03) and for medullary lesions with respiratory depression (*p* = 0.01) (Figure 3D).

### 3.4 Heteroplasmy Level‐Phenotype Correlation

**FIGURE 4:**
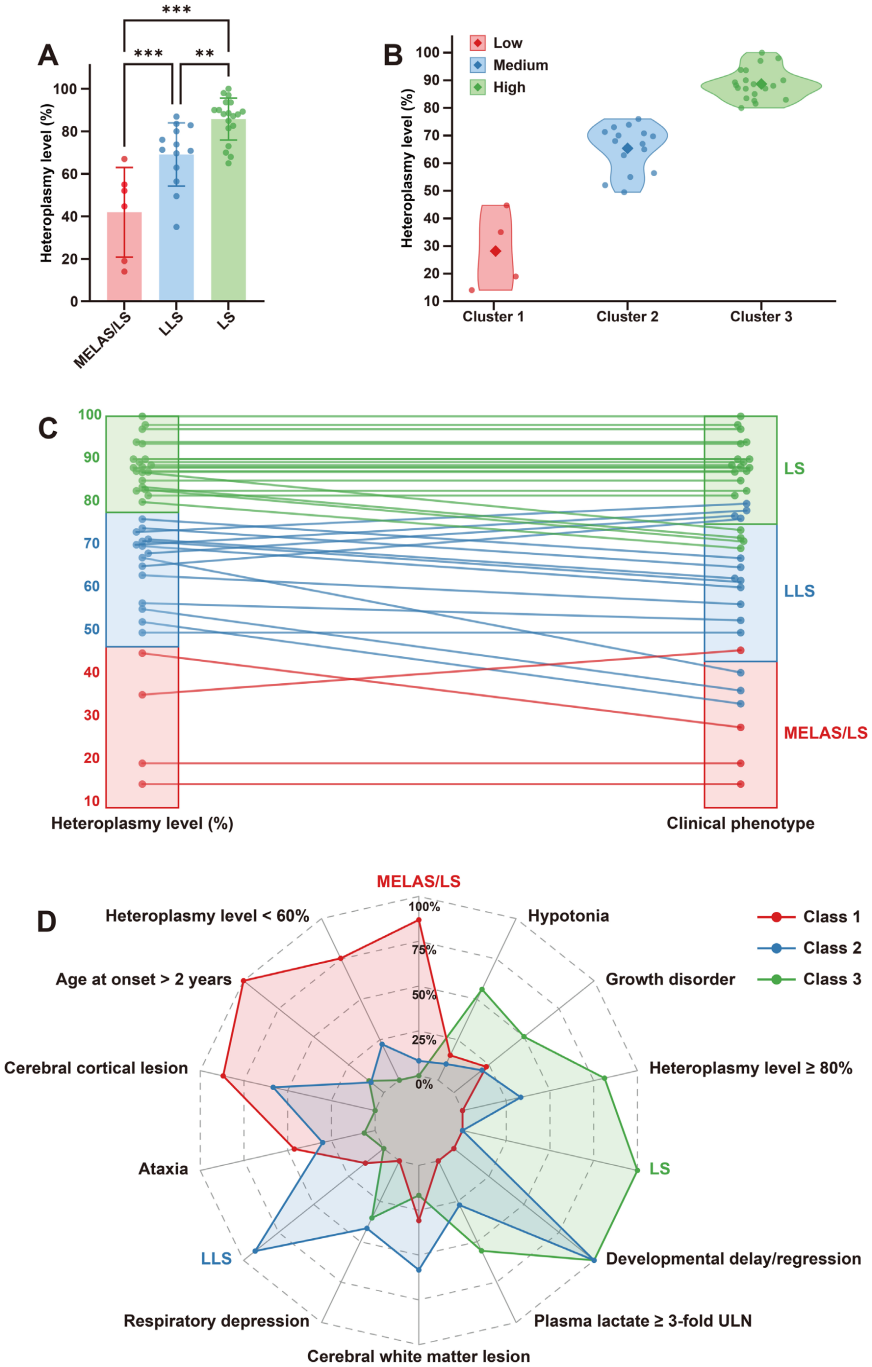
Heteroplasmy‐phenotype correlation in patients with the m.10191 T > C mutation. (A) Comparison of heteroplasmy level across clinical phenotypes (LS, LLS, MELAS/LS). Data are presented as mean ± standard deviation; Asterisks indicate statistically significant (Bonferroni correction, *p < 0.05, **p < 0.01, ***p < 0.001). (B) Clustering of patients into high‐, medium‐, and low‐heteroplasmy level groups based on k‐means clustering. (C) Cross‐analysis of classification consistency between heteroplasmy level clusters and clinical phenotypic groups. (D) Characteristic profiles of three latent classes identified through latent class analysis. LLS, Leigh‐like syndrome; LS, Leigh syndrome; MELAS, mitochondrial encephalomyopathy with lactate acidosis and stroke‐like episodes; MELAS/LS, MELAS/LS overlap syndrome; ULN, upper limit of normal.

Among the 39 patients with available data, the median heteroplasmy level was 80.0% (IQR 23.2%; range 14.0%–100.0%). Heteroplasmy level differed significantly across LS, LLS, and MELAS/LS groups (*p* < 0.001). Specifically, the LS group exhibited the highest heteroplasmy level (85.8% ± 9.9%), which was significantly higher than that in the LLS group (69.1% ± 14.9%; Bonferroni‐corrected *p* = 0.005) and the MELAS/LS group (42.0% ± 21.0%; Bonferroni‐corrected *p* < 0.001). The LLS group also had a significantly higher heteroplasmy level than the MELAS/LS group (Bonferroni‐corrected *p* < 0.001) (Figure 4A). Based on clustering analysis, patients were categorized into high‐, medium‐, and low‐level subgroups, with mean heteroplasmy levels of 88.7% ± 5.5%, 65.4% ± 8.4%, and 28.2% ± 14.2%, respectively (Figure 4B). Cross‐tabulation of heteroplasmy level clusters against clinical phenotypic groups revealed cluster purities of 80.0%, 53.3%, and 75.0% for the high, medium, and low heteroplasmy level groups. Corresponding correct classification rates for LS, LLS, and MELAS/LS phenotypic groups were 80.0%, 61.5%, and 50.0%, respectively, yielding an overall correct classification rate of 69.2% (Figure 4C). Heteroplasmy level distribution differed significantly across clinical phenotypic groups (*p* < 0.001, Fisher's exact test). The proportion of high heteroplasmy level was significantly higher in the LS group (80.0%) than in the LLS (30.8%) and MELAS/LS groups (0%); the proportion of low heteroplasmy level was significantly higher in the MELAS/LS group (50.0%) than in the LLS (7.7%) and LS groups (0%); and the proportion of medium heteroplasmy level differed significantly between the LS and LLS groups (20.0% vs. 61.5%) (all Bonferroni‐corrected *p* < 0.05). These results collectively support a correlation between heteroplasmy level and specific clinical phenotypes.

Prior to LCA, clinical manifestations with potential phenotypic discriminative value were prescreened. Results showed significant between‐group differences in age at onset, heteroplasmy level, and developmental delay/regression (FDR‐corrected *p* < 0.001, = 0.008, and < 0.001, respectively). Nine clinical manifestations showing between‐group differences (uncorrected *p* < 0.05) were selected for subsequent LCA (Table S2). LCA identified a three‐class solution as optimal (LMR‐LRT *p* = 0.05, BLRT *p* < 0.001, AIC = 568.33, BIC = 653.24), outperforming two‐ and four‐class models (Table S3). A radar chart was generated based on conditional probabilities (Table S4) of each characteristic across the latent classes to visualize their characteristic profiles. The three latent classes showed correspondence with the clinical phenotypes of MELAS/LS, LLS, and LS (conditional probabilities: 0.869, 0.917, and 1.000, respectively). The profiles of each class were as follows: Class 1 (corresponding to MELAS/LS) was characterized predominantly by low heteroplasmy level, age at onset > 2 years, ataxia, and cortical lesions; Class 2 (corresponding to LLS) by medium heteroplasmy level, respiratory depression, and white matter lesions; and Class 3 (corresponding to LS) by high heteroplasmy level, developmental delay/regression, growth disorder, hypotonia, and plasma lactate ≥ 3‐fold ULN (Figure 4D). To validate the stability of the LCA three‐class solution, a two‐step cluster analysis using complete data analysis was performed, confirming the three‐cluster solution. The cluster characteristics were broadly consistent with the LCA classes (Table S5).

### 3.5 Disease Course and Prognosis

**FIGURE 5:**
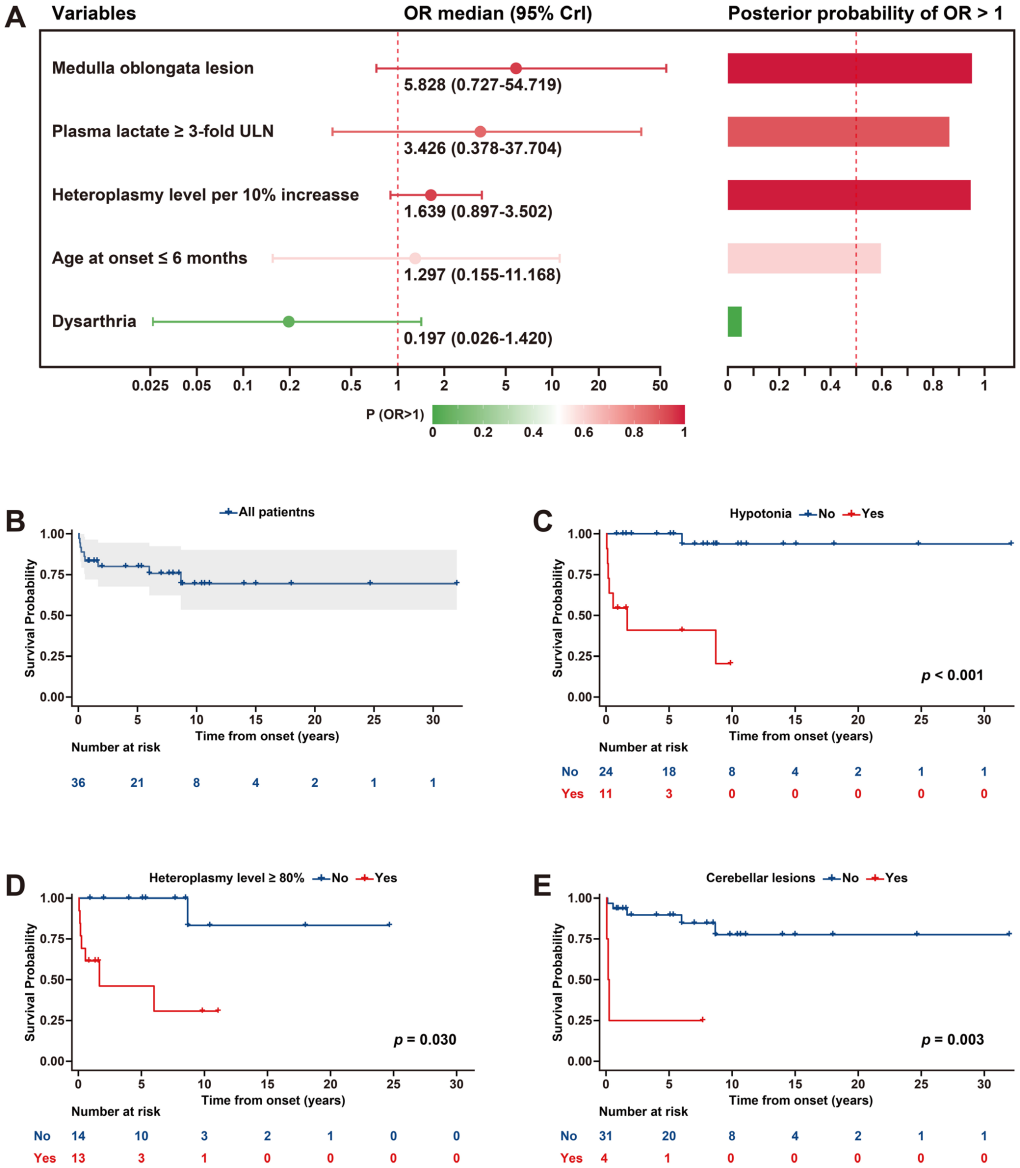
Prognostic factors and survival analysis in patients with the m.10191 T > C mutation. (A) Multivariable Bayesian logistic regression of factors associated with disease progression. (B) Overall Kaplan–Meier survival curve for all patients (*n* = 36). (C–E) Kaplan–Meier survival curves stratified by the presence or absence of (C) hypotonia, (D) heteroplasmy level ≥ 80%, and (E) cerebellar lesions. All Log‐rank *p* values are false discovery rate‐corrected. CrI, credible interval; OR, odds ratio; ULN, upper limit of normal.

Among the 38 patients with assessable prognostic information, 3 (7.9%) showed clinical improvement, 8 (21.0%) remained stable, and 27 (71.1%) experienced disease progression. We first performed a univariate analysis by grouping patients according to progression status to preliminarily identify factors associated with prognosis (Table S6). Variables with uncorrected *p* < 0.10—including age at onset < 6 months, heteroplasmy level, dysarthria, plasma lactate ≥ 3‐fold ULN, and medulla oblongata lesions—were incorporated into the multivariable Bayesian logistic regression. To avoid multicollinearity, respiratory depression, though significant in univariate analysis, was not included due to its high correlation with medulla lesions (Fisher's exact test *p* = 0.002, Phi = 0.55). Bayesian logistic regression identified medulla lesions (*P* (OR > 1) = 0.95) and high heteroplasmy level (*P* (OR > 1) = 0.95) as strong risk factors for disease progression, whereas dysarthria (*P* (OR > 1) = 0.05) was a strong protective factor. Plasma lactate ≥ 3‐fold ULN showed a moderate‐risk tendency (*P* (OR > 1) = 0.86) (Figure 5A). Owing to the limited sample size, however, the posterior distributions of the ORs were diffuse (e.g., medulla lesions: median OR = 5.83, 95% CrI: 0.73–54.72), indicating uncertainty in the magnitude of the associations despite clear directional evidence. Therefore, these prognostic findings should be considered exploratory and interpreted with caution.

Among 36 patients with available survival information, 27 (75.0%) were alive, and 9 (25.0%) had died at the last follow‐up or per the most recent literature report. Of the 9 deceased patients, 8 were diagnosed with LS and 1 with nonspecific MD; the median age at death was 1.00 year (IQR 5.19), and the median disease duration was 0.50 years (IQR 3.69). Kaplan–Meier analysis of all patients (*n* = 36) yielded 1‐, 5‐, and 10 year survival rates of 83.3% (95% CI: 72.0%–96.4%), 80.0% (95% CI: 67.7%–94.5%), and 69.5% (95% CI: 53.5%–90.2%), respectively (Figure 5B). Further subgroup analyses stratified by clinical, metabolic, and imaging features (all log‐rank *p* values are provided in Table S7) revealed that hypotonia (FDR‐corrected *p* < 0.001), heteroplasmy level ≥ 80% (FDR‐corrected *p* = 0.030), and cerebellar lesions (FDR‐corrected *p* = 0.003) were significantly associated with poorer survival (Figure 5C–E).

### 3.6 Sensitivity Analysis

First, in the analysis restricted to the newly recruited cohort (*n* = 14), pairwise association analysis of neuroimaging lesions showed trends consistent with the primary analysis (Figure S1). However, co‐occurrence of globus pallidus and midbrain/pons lesions did not reach statistical significance due to the limited sample size. The heteroplasmy‐phenotype correlation remained consistent with the primary findings, achieving an overall correct classification rate of 64.3% (Figure S2). Prognostic analysis indicated that factors such as heteroplasmy level, age at onset < 6 months, severe hyperlactatemia, medullary involvement, and dysarthria were associated with disease progression. Although the latter four factors did not reach statistical significance in this smaller subset, they exhibited the largest between‐group differences (Table S8). Survival analysis identified hypotonia, heteroplasmy level ≥ 80%, and cerebellar lesions as associated with poorer survival. Among these, the latter two factors did not achieve FDR‐corrected significance but consistently showed the largest between‐group differences (Table S9). Second, analyses restricted to blood‐derived heteroplasmy data (*n* = 33) confirmed that the heteroplasmy‐phenotype correlation remained highly significant (Figure S3). High heteroplasmy levels were associated with both disease progression and poorer survival, consistent with primary analysis findings (Figure S4). Detailed results are provided in Appendix S2.

## 4 Discussion

By integrating 14 newly ascertained cases with 38 previously reported cases, we assembled the largest cohort to date of patients carrying the m.10191 T > C mutation. This expanded dataset delineates a continuous yet heterogeneous disease spectrum, ranging from typical LS to MELAS‐like presentations with SLEs. CI deficiency caused by mitochondrial DNA (mtDNA) mutations can lead to widespread multisystem damage [39]. Correspondingly, the m.10191 T > C mutation in this study also demonstrated extensive multisystem involvement, with over three‐quarters of patients exhibiting dysfunction in three or more organ systems, predominantly affecting the neurological and metabolic systems. The clinical presentation demonstrates considerable heterogeneity, with seizures, developmental delay, and dystonia predominating. Epilepsy affected more than 80% of patients in our cohort, far exceeding the 20%–60% prevalence typically reported in LS and general mitochondrial disease cohorts [40, 41]. Seizure types were diverse, including focal, generalized tonic–clonic, and myoclonic seizures. Furthermore, prior studies had reported that patients carrying the m.10191 T > C mutation may also present with Lennox–Gastaut syndrome (LGS) or refractory status epilepticus [8, 10], collectively underscoring epilepsy as a prominent and distinctive hallmark of this mutation. Mechanistically, epileptogenesis in primary mitochondrial disorders is mainly attributed to ATP depletion secondary to defective oxidative phosphorylation (OXPHOS), particularly in the context of complex I and IV deficiencies [42]. However, the precise molecular and electrophysiological mechanisms conferring epilepsy susceptibility specific to the m.10191 T > C mutation require further elucidation. Abnormal muscle tone was also frequently observed, affecting nearly 70% of patients (29/43). Patients with infantile onset typically exhibited hypotonia, whereas hypertonia was more commonly seen in older individuals or emerged progressively with age. Notably, in contrast to typical LS cohorts, in which hypotonia predominates (74.6%) [43], patients carrying the m.10191 T > C mutation showed a relatively lower proportion of hypotonia (30.2%) and a higher proportion of dystonia or spasticity. This difference likely reflects the phenotypic heterogeneity arising from the coexistence of LS and non‐LS phenotypes within this mutation‐specific cohort. Developmental delay or regression constituted the most common initial symptom, aligning with the predominance of LS and LLS phenotypes in our cohort. In contrast, despite its high overall prevalence, epilepsy less often served as the presenting symptom—a pattern commonly associated with non‐LS phenotypes—suggesting that seizures tend to emerge during the disease course.

We systematically delineated the neuroimaging spectrum associated with the m.10191 T > C mutation. Overall, imaging findings were concordant with the heterogeneity of clinical phenotypes and did not demonstrate a specific genotype‐imaging enrichment pattern, in contrast to those reported for certain other genes (e.g., medullary predilection in *SURF1* deficiency [41]). A prior retrospective MRI study of 30 patients with CI deficiency reported involvement of the brainstem and/or basal ganglia—most prominently affecting the putamen—in over 90% of cases [44]. Our findings closely align with these observations, likewise demonstrating frequent involvement of the basal ganglia and/or brainstem, with the putamen being a commonly affected site. Notably, we observed co‐occurrence of lesions in the globus pallidus and midbrain, as well as in the cerebellum and medulla. This pattern may suggest shared anatomical or physiological vulnerabilities in these regions, potentially implying a selective impact of the mutation on specific neural pathways or networks rather than random damage to individual nuclei.

Heteroplasmy levels in mtDNA mutations are a key determinant of cellular phenotype (i.e., whether respiratory dysfunction occurs) and is generally considered to directly influence the phenotypic variability in mtDNA‐related diseases [16, 45]. Previous studies have established clear genotype–phenotype correlations for certain pathogenic variants. For example, the m.8993 T > G mutation in MT‐ATP6 manifests as neuropathy, ataxia, and retinitis pigmentosa (NARP) at heteroplasmy levels of 70%–90%, whereas levels > 90% are typically associated with LS phenotype [46]. Similarly, in a pedigree carrying the m.8363G > A mutation in *MTTK*, lower heteroplasmy levels were linked to adult‐onset myoclonic epilepsy with ragged red fibers (MERRF), whereas near‐homoplasmic levels led to LS [47]. In this study, we demonstrate for the first time that the m.10191 T > C mutation exhibits a clear heteroplasmy‐phenotype correlation: lower heteroplasmy levels were associated with MELAS‐like presentations, whereas higher heteroplasmy levels correlated with classical LS. LCA further corroborates this pattern, identifying three latent classes that closely corresponded to MELAS/LS, LLS, and LS phenotypes, each characterized by distinct heteroplasmy level ranges and characteristic combinations of clinical and neuroimaging features. This data‐driven classification provides model‐based statistical validation for the close association between heteroplasmy level and clinical presentation. The severity of energy deficiency likely contributes to the ultimate clinical expression of the molecular defect. Compared with other mitochondrial disorders, such as MERRF or MELAS, LS may reflect a more severe ATP deficit [48]. Our findings support positioning LS at the severe end of the disease spectrum for this mutation, consistent with the more profound energy failure expected at high heteroplasmy levels. However, it should be noted that heteroplasmy levels do not always strictly correspond to clinical phenotypes. Indeed, many other mtDNA mutations lack clear heteroplasmy‐phenotype correlations. Even within this study, heteroplasmy alone did not fully predict phenotype in every case, achieving an overall classification accuracy of approximately 70%. This partial inconsistency can be partly attributed to our phenotypic classification scheme, distinguishing between classical LS and LLS, with most misclassifications occurring within the LLS subgroup. Recently, the term “Leigh syndrome spectrum (LSS)” was proposed to integrate LS and LLS into a single continuum [49]. When LS and LLS were combined into a unified LSS category and analyzed in a two‐cluster analysis, classification accuracies improved significantly (90.9% for LSS and 83.3% for MELAS/LS), resulting in an overall accuracy of 89.7%. Furthermore, the imperfect correlation between heteroplasmy level and phenotype might be attributable to modulating influences of nuclear genetic background, environmental factors, and epigenetic regulation, which collectively obscure this association at the individual level [45]. Additionally, measuring heteroplasmy levels in peripheral blood rather than target tissues, such as the brain, may attenuate the observed correlation. Although brain tissues were unavailable for analysis, we compared heteroplasmy levels across different tissues (blood, muscle, fibroblasts) in patients with multi‐tissue data (Appendix S3). In the LS phenotype, mean heteroplasmy levels in fibroblasts tended to be lower compared to those in blood and muscle, although these differences were not statistically significant. In non‐LS phenotypes, muscle heteroplasmy levels were higher than those in blood and fibroblasts, but statistical comparisons were limited by the small sample size. Furthermore, tissue‐specific variability in heteroplasmy levels differed; fibroblasts exhibited higher variability (e.g., in LS phenotype, coefficient of variation (CV) = 28.0%), whereas blood and muscle showed lower variability (blood CV = 11.9%; muscle CV = 13.1%) (Figure S5). These observations indicate that heteroplasmy measurements in target tissues might correlate more closely with clinical phenotypes, and that heteroplasmy stability may vary across tissue types. Therefore, stronger heteroplasmy‐phenotype correlations might emerge if measured directly in relevant target tissues.

Our study provides a comprehensive characterization of disease course and prognostic features in patients carrying the m.10191 T > C mutation. While the course in most patients aligns with the natural history of LS, the presence of MELAS/LS phenotypes adds complexity to disease management. Disease progression was observed in over 70% of patients, with medullary involvement, severe hyperlactatemia (plasma lactate ≥ 3‐fold ULN), and high heteroplasmy level correlating with clinical deterioration. Medullary lesions were directly associated with respiratory insufficiency or failure, whereas pronounced hyperlactatemia frequently indicated critical illness or extensive mitochondrial energy metabolism dysfunction, both representing robust indicators of poor prognosis [50]. In our study, dysarthria showed a negative association with disease progression. This finding is primarily attributable to the fact that dysarthria is a clinical manifestation associated with late‐onset cases, which generally have a better prognosis compared with onset before 6 months of age. In our cohort, the prevalence of dysarthria differed significantly between early‐onset (< 6 months, *n* = 14) and late‐onset (> 2 years, *n* = 16) patients (1/14 [7.1%] vs. 7/16 [43.8%], Fisher's exact test *p* = 0.039). This is consistent with a previous large cohort study of LS, which reported that dysarthria was completely absent in early‐onset LS (0/55) but enriched in late‐onset LS (21/72), and further prognostic analysis showed that dysarthria was associated with significantly better survival [41]. Therefore, dysarthria likely serves as a clinical marker of late‐onset, slower‐progressive phenotypes rather than a direct biological protective factor. The limited sample size and wide credible intervals further caution against overinterpretation. The overall mortality rate in our cohort was 25%, confined to patients within the LS phenotype (including one case classified as nonspecific MD). Notably, the 5 year survival rate reached 80%, markedly exceeding previously reported overall outcomes for CI deficiency, where median survival age is 10 months, and over 75% of patients die before age 10 [51]. Even when restricted to LS or LLS phenotypes, long‐term survival remained favorable, with a 5 year survival rate of 75.7% and a median survival of 4.54 years (IQR 9.08), outperforming historical data from large LS cohorts [41, 43]. A previously reported LS patient with this mutation even survived beyond 25 years [9]. Cybrid experiments indicate that, compared with other CI pathogenic mutations, m.10191 T > C retains partial residual CI activity (30%–40%) without triggering severe mitochondrial network fragmentation or quality control activation [20, 52], likely preserving basal energy output and providing a critical survival buffer. Subgroup survival analysis further identified heteroplasmy level ≥ 80%, hypotonia, and cerebellar lesions as significant predictors of reduced survival. High heteroplasmy level and hypotonia, both closely linked to classical LS phenotypes, were thus associated with poorer survival. Cerebellar abnormalities, which often indicate more extensive and severe posterior fossa involvement (frequently accompanied by medullary lesions), may further heighten the mortality risk. Early identification and intervention based on these risk factors may mitigate complications, improve quality of life, and prolong survival.

## 5 Limitations

The present study has several limitations. First, data from literature‐derived cases were limited to secondary sources, introducing incomplete reporting and variability in collection standards, as well as potential publication and selection bias that may skew the estimated disease spectrum and prognosis. Second, despite constituting the largest cohort to date, the overall sample size remains relatively limited, with uneven phenotypic distribution (e.g., very few MELAS‐like cases), constraining our ability to perform robust statistical inferences. Third, heteroplasmy level data for a minority of patients (*n* = 6) were derived from different tissues (muscle or fibroblasts); given tissue‐specific heteroplasmy variability, this may have attenuated the consistency of the observed correlation between heteroplasmy and phenotype/prognosis. Fourth, neuroimaging features were primarily extracted from descriptive reports rather than original image data, limiting analyses to approximate lesion localization for preliminary statistical associations and precluding detailed evaluation of lesion morphology, progression, or quantitative characteristics. Finally, the Bayesian logistic regression analysis was limited by the small sample size, resulting in wide credible intervals and low precision of the effect estimates. These findings should therefore be considered exploratory and require validation in larger independent cohorts.

## 6 Conclusion

This study presents the largest cohort of patients with the m.10191 T > C mutation to date, delineating a continuous spectrum from LS to MELAS‐like phenotypes and systematically characterizing clinical manifestations, neuroimaging features, heteroplasmy–phenotype correlations, and prognostic factors. These findings provide important insights for understanding the phenotypic variability of the m.10191 T > C mutation, optimizing diagnostic pathways, and advancing individualized prognostic assessment.

## Author Contributions

(i) Zimeng He and Fang contributed to the conception and design of the study. (ii) Zimeng He, Huafang Jiang, Tongyue Li, Yang Liu, Zhimei Liu, Danmin Shen, Ruoyu Duan, Ying Zou, Chaolong Xu, Minhan Song, Yunxi Zhang, Zixuan Zhang, Rui Shen, Mingxi Sun, and Hong Xue contributed to the acquisition, analysis, and interpretation of data. iii. Zimeng He and Fang contributed to drafting the article or revising it critically for important intellectual content.

## Funding

This study was supported by the National Natural Science Foundation of China (82271493), Beijing Municipal Science & Technology Commission, Administrative Commission of Zhongguancun Science Park (Z241100007724008), Science and Technology Developmental Project of Weifang (Medicine) (2023YX061).

## Ethics Statement

This study was approved by the Ethics Committee of Beijing Children's Hospital, affiliated with Capital Medical University ([2022]‐E‐121Y), and written informed consent was obtained from the parent or legal guardian of every participant.

We confirm that we have read the Journal's position on issues involved in ethical publication and affirm that this report is consistent with those guidelines.

## Conflicts of Interest

The authors declare no conflicts of interest.

## Supporting information


**Appendix S1:** Literature review methodology.

**Appendix S2:** Sensitivity analysis.
**Figure S1:** Neuroimaging findings in the newly recruited cohort (*n* = 14).
**Figure S2:** Heteroplasmy‐phenotype correlation in the newly recruited cohort (*n* = 14).
**Table S8:** Comparison of characteristics between patients with and without disease progression in the newly recruited cohort (*n* = 14).
**Table S9:** Results of log‐rank tests for subgroup survival analyses in the newly recruited cohort (*n* = 14).
**Figure S3:** Heteroplasmy‐phenotype correlation restricted to blood‐derived heteroplasmy data (*n* = 33).
**Figure S4:** Prognostic factors and survival analysis restricted to blood‐derived heteroplasmy data (*n* = 33).

**Appendix S3:** Multi‐tissue comparison of heteroplasmy levels in patients with the m.10191 T > C mutation.
**Figure S5:** Multi‐tissue comparison of heteroplasmy levels in patients with the m.10191 T > C mutation. (A) Heatmap of heteroplasmy levels across multiple tissues (blood, muscle, urine, and fibroblasts) in 13 patients with multi‐tissue data. Each column represents one patient, with patient numbers corresponding to those in the main text. Black numbers indicate patients derived from the literature, and green numbers indicate patients from the newly recruited cohort. Patients are grouped by phenotype (LS, *n* = 8; LLS, *n* = 3; MELAS/LS, *n* = 1; MELAS‐like, *n* = 1). Color intensity (blue to red) reflects heteroplasmy level (%). Missing data are indicated in gray (B–D). Comparison of heteroplasmy levels across tissue sources by phenotype. (B) LS phenotype (blood: *n* = 15; muscle: *n* = 7; fibroblasts: *n* = 4). One‐way ANOVA *p* = 0.080. (C) LLS phenotype (blood: *n* = 12; muscle: *n* = 2). (D) MELAS/LS and MELAS‐like phenotypes combined (blood: *n* = 6; muscle: *n* = 1; fibroblasts: *n* = 1). Data are presented as mean ± SD. Statistical comparisons were not performed for (C) and (D) due to small sample sizes. Abbreviations: LS, Leigh syndrome; LLS, Leigh‐like syndrome; MELAS, mitochondrial encephalomyopathy with lactate acidosis and stroke‐like episodes; MELAS/LS, MELAS/LS overlap syndrome.

**Table S1:** Characteristics of the 38 patients with the m.10191 T > C mutation identified from the literature.
**Table S2:** Comparison of characteristics between patients with and without cortical involvement.
**Table S3:** Fit indices for two‐ to four‐class latent class analysis models.
**Table S4:** Probability of each variable across the three latent classes.
**Table S5:** Clinical characteristics of the three clusters identified by two‐step cluster analysis (*n* = 28).
**Table S6:** Comparison of characteristics between patients with and without disease progression.
**Table S7:** Results of log‐rank tests for subgroup survival analyses.

## Data Availability

The data that support the findings of this study are available from the corresponding author upon reasonable request.
