# Supplementary material for: Clinical Spectrum, Heteroplasmy‐Phenotype Correlation, and Prognosis of the MT‐ND3 m.10191 T > C Mutation
Source: CNS Neurosci Ther. 2026 Jun 19;32(6):e70997. doi: 10.1002/cns.70997 (PMC13280565; doi:10.1002/cns.70997)
Supplement: Supplementary file 1 — Appendix S1: Literature review methodology. [file CNS-32-e70997-s001.docx]

**Appendix S1**

**Literature review methodology**

**Literature review method**

A systematic review was conducted following the Preferred Reporting Items for Systematic Reviews and Meta-Analyses of individual participant data (PRISMA‑IPD) guidelines^[1]^. A comprehensive search was performed in PubMed, EMBASE, and ClinVar databases without language restrictions, using the following terms individually or in combination: “10191T>C”, “10191 mutation”, “10191 T mutation”, “mitochondrial 10191”, “m.10191”, “MT‑ND3 10191”. Additionally, reference lists of included articles were manually searched for potentially missed publications.

**Inclusion criteria:** Articles were eligible for inclusion if they: (1) reported one or more patients with a genetically confirmed m.10191T>C mutation; (2) provided individual‑level clinical information (at least demographic data or clinical manifestations); and (3) were published in peer‑reviewed journals. Articles reporting only aggregated data were included only if individual‑level patient information was extractable.

**Exclusion criteria:** Cases were excluded if: (1) the m.10191T>C mutation was not confirmed by genetic testing; (2) the same patient appeared in multiple publications (in which case the most complete report was retained, and duplicates were excluded); or (3) insufficient clinical information was available to determine at least the phenotype category.

**Data extraction:** The extracted data included first author, publication year, sex, age at onset, initial symptom, heteroplasmy level, clinical manifestations, neuroimaging features, treatment, outcome, and other relevant information. For cases with incomplete data, variables that could not be ascertained from the published reports were coded as “unknown” and excluded from the corresponding analyses.

**Search strategies in ClinVar, PubMed and EMBASE**

| ClinVar | 10191T>C |
| --- | --- |
| PubMed | (((((10191T>C) OR (m.10191)) OR (10191 mutation)) OR (10191 T mutation)) OR (mitochondrial 10191)) OR (MT-ND3 10191) |
| EMBASE | ('10191T>C'/exp OR 'm.10191'/exp OR '10191 mutation'/exp OR '10191 T mutation'/exp OR 'mitochondrial 10191'/exp OR 'MT-ND3 10191'/exp) |

**List of the studies excluded because of duplicate patient reporting**

| **Authors** | **Title** | **Journal** | **PMID** |
| --- | --- | --- | --- |
| Antozzi C, Franceschetti S, Filippini G, et al. | Epilepsia partialis continua associated with NADH-coenzyme Q reductase deficiency | Journal of the neurological sciences | 7608730 |
| Ma YY, Wu TF, Liu YP, et al. | Mitochondrial respiratory chain enzyme assay and DNA analysis in peripheral blood leukocytes for the etiological study of Chinese children with Leigh syndrome due to complex I deficiency | Mitochondrial DNA | 22947169 |
| Wojtala A, Karkucinska-Wieckowska A, Sardao VA, et al. | Modulation of mitochondrial dysfunction-related oxidative stress in fibroblasts of patients with Leigh syndrome by inhibition of prooxidative p66Shc pathway | Mitochondrion | 28739512 |
| Newstead SM, Finsterer J. | Metabolic inflexibility and unusual catabolism in Leigh-like syndrome due to m.10191T>C | Clinical nutrition ESPEN | 37344065 |
| Newstead SM, Finsterer J. | Hemolytic Anemia Requiring Splenectomy in Leigh-Like Syndrome due to the Variant m.10191T>C in MT-ND3 | Journal of hematology | 37692867 |
| Newstead SM, Scorza CA, Fiorini AC, et al. | Mitochondrial small fiber neuropathy as a novel phenotypic trait of Leigh-like syndrome due to the variant m.10191T>C in MT-ND3 | Clinics (Sao Paulo) | 37196589 |
| Newstead SM, Finsterer J. | Hyperkinesias in Leigh-like Syndrome with Complex-I Deficiency Due to m.10191T>C in MT-ND3 | Annals of African medicine | 39034583 |

**References**

[1] Stewart L A, Clarke M, Rovers M, et al. Preferred Reporting Items for Systematic Review and Meta-Analyses of individual participant data: the PRISMA-IPD Statement [J]. Jama, 2015, 313(16): 1657-65.
