# Supplementary material for: Clinical Spectrum, Heteroplasmy‐Phenotype Correlation, and Prognosis of the MT‐ND3 m.10191 T > C Mutation
Source: CNS Neurosci Ther. 2026 Jun 19;32(6):e70997. doi: 10.1002/cns.70997 (PMC13280565; doi:10.1002/cns.70997)
Supplement: Supplementary file 2 — Appendix S2: Sensitivity analysis. Figure S1: Neuroimaging findings in the newly recruited cohort (n = 14). Figure S2: Heteroplasmy‐phenotype correlation in the newly recruited cohort (n = 14). Table S8: Comparison of characteristics between patients with and without disease progression in the newly recruited cohort (n = 14). Table S9: Results of log‐rank tests for subgroup survival analyses in the newly recruited cohort (n = 14). Figure S3: Heteroplasmy‐phenotype correlation restricted to blood‐derived heteroplasmy data (n = 33). Figure S4: Prognostic factors and survival analysis restricted to blood‐derived heteroplasmy data (n = 33). [file CNS-32-e70997-s004.docx]

**Appendix S2**

**Sensitivity analysis**

***Section 1 Sensitivity analysis restricted to the newly recruited cohort (n = 14)***

**Neuroimaging findings**

Basal ganglia and brainstem lesions co-occurred in nine patients, and thalamic lesions co-occurred with brainstem abnormalities in seven patients (Figure S1A). Pairwise association analysis demonstrated co-occurrence of putamen and caudate nucleus involvement (Phi = 1.00, uncorrected *p* < 0.01, FDR-corrected *p* = 0.02). Although not statistically significant, associations were also observed between the globus pallidus and midbrain/pons lesions (Phi = 0.60, uncorrected *p* = 0.05, FDR-corrected *p* = 0.76), as well as between medullary and cerebellar lesions (Phi = 0.65, uncorrected *p* = 0.07, FDR-corrected *p* = 0.76) (Figure S1B).


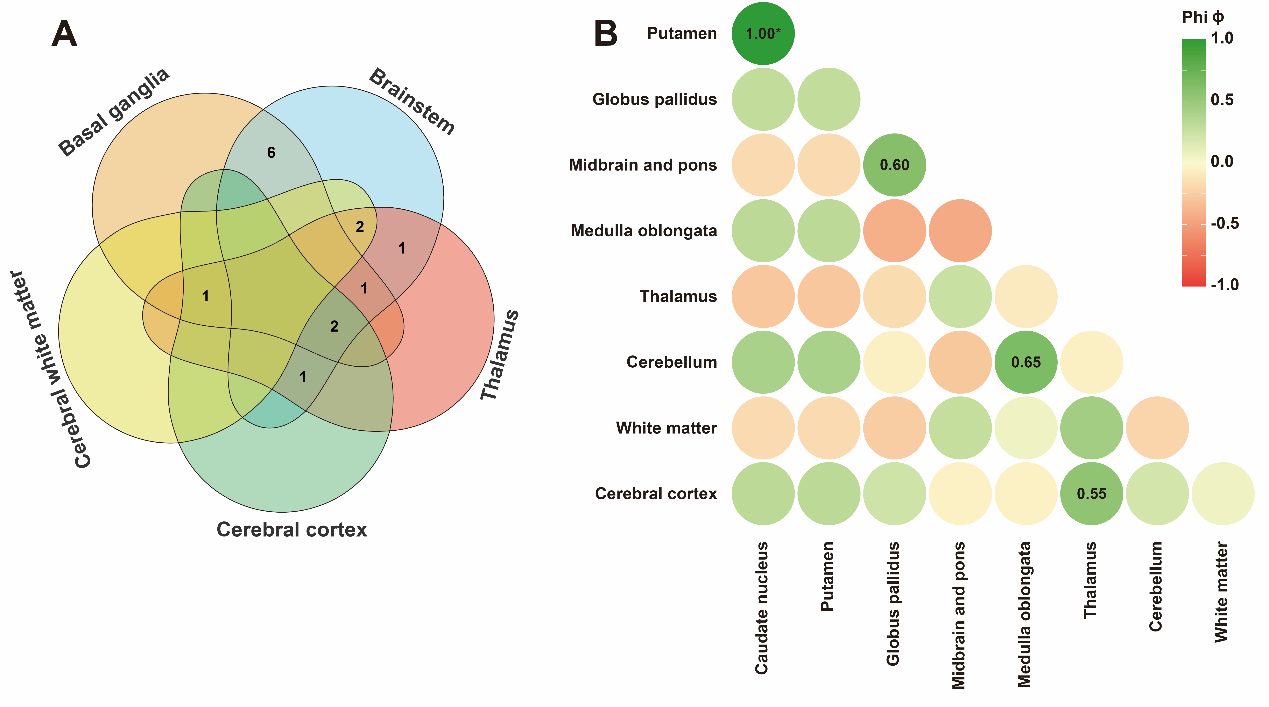


**Figure S1** Neuroimaging findings in the newly recruited cohort (n = 14)

**(A)** Overlap of lesions among five major brain structures. **(B)** Pairwise association between specific lesion locations. Associations were quantified using the Phi coefficient and visualized in a correlation matrix. Green represents a positive association. Asterisks indicate statistically significant correlations (False discovery rate correction, ^*^*p* < 0.05).

**Heteroplasmy level-phenotype correlation**

Among the 14 patients, the mean heteroplasmy level was 74.7% ± 15.4%. The LS group had the highest heteroplasmy level (86.5% ± 7.9%), significantly higher than that in the LLS group (71.0% ± 12.6%; Bonferroni-corrected *p* = 0.033). The single MELAS/LS patient had a heteroplasmy level of 44.7%, which was lower than those in the LS and LLS groups; statistical comparisons involving this group were not performed due to the small sample size (Figure S2A). Based on clustering analysis, patients were categorized into high-, medium-, and low-level subgroups, with mean heteroplasmy levels of 88.3% ± 3.3%, 73.0% ± 2.1%, and 50.2% ± 5.9%, respectively (Figure S2B). Cross-tabulation of heteroplasmy level clusters against clinical phenotypic groups revealed cluster purities of 66.7%, 80.0%, and 33.3% for the high, medium, and low heteroplasmy level groups. Corresponding correct classification rates for LS, LLS, and MELAS/LS phenotypic groups were 80.0%, 50.0%, and 100.0%, respectively, yielding an overall correct classification rate of 64.3%.


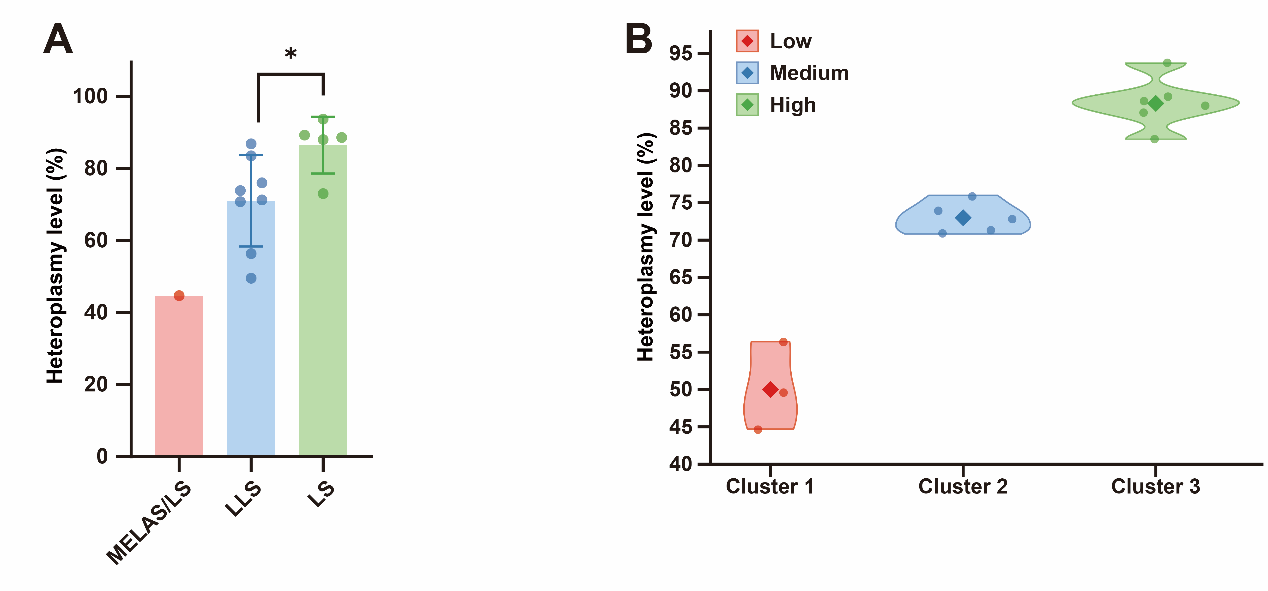


**Figure S2** Heteroplasmy-phenotype correlation in the newly recruited cohort (n = 14)

**(A)** Comparison of heteroplasmy level across clinical phenotypes (LS, LLS, MELAS/LS). Data are presented as mean ± standard deviation; Asterisks indicate statistically significant (Bonferroni correction, ^*^*p* < 0.05). **(B)** Clustering of patients into high-, medium-, and low-heteroplasmy level groups based on k‑means clustering. **Abbreviations:** LS, Leigh syndrome; LLS, Leigh-like syndrome; MELAS, mitochondrial encephalomyopathy with lactate acidosis and stroke-like episodes; MELAS/LS, MELAS/LS overlap syndrome.

**Disease course and prognosis**

Univariate analysis of prognostic factors in the newly recruited cohort showed that heteroplasmy level ≥ 80% (uncorrected *p* = 0.03), severe hyperlactatemia (uncorrected *p* = 0.09), dysarthria (uncorrected *p* = 0.09), age at onset < 6 months (uncorrected *p* = 0.22), and medullary involvement (uncorrected *p* = 0.22) remained the five factors with the greatest prognostic discriminatory value, consistent with the primary analysis. Detailed between‑group comparisons are presented in Table S8.

Subgroup survival analysis in the newly recruited cohort showed that hypotonia (uncorrected *p* < 0.01, FDR-corrected *p* = 0.04), heteroplasmy level ≥ 80% (uncorrected *p* = 0.04, FDR-corrected *p* = 0.59), and cerebellar lesions (uncorrected *p* = 0.11, FDR-corrected *p* = 0.75) were the three factors most strongly associated with survival outcomes, consistent with the primary analysis. Detailed between‑group comparisons are presented in Table S9.

**Table S8** Comparison of characteristics between patients with and without disease progression in the newly recruited cohort (n = 14)

| **Characteristics** | **Progression** | **Non-progression** | **Uncorrected *p* value** |
| --- | --- | --- | --- |
| Male | 8/9 (88.89%) | 3/5 (60.00%) | 0.505 |
| Onset age < 6m | 4/9 (44.44%) | 0/5 (0.00%) | 0.221 |
| Onset age > 2y | 2/9 (22.22%) | 2/5 (40.00%) | 0.580 |
| Heteroplasmy level ≥ 80% | 6/9 (66.67%) | 0/5 (0.00%) | 0.031^*^ |
| Maternal | 3/9 (33.33%) | 2/5 (40.00%) | 1.000 |
| Migraine | 0/9 (0.00%) | 1/5 (20.00%) | 0.357 |
| Stroke-like episodes | 0/9 (0.00%) | 1/5 (20.00%) | 0.357 |
| Dev delay/regression | 8/9 (88.89%) | 3/5 (60.00%) | 0.505 |
| Cognitive impairment | 6/8 (75.00%) | 3/5 (60.00%) | 1.000 |
| Seizure | 7/9 (77.78%) | 2/5 (40.00%) | 0.266 |
| Myoclonus | 0/9 (0.00%) | 0/5 (0.00%) | NA |
| Hypertonia | 5/9 (55.56%) | 3/5 (60.00%) | 1.000 |
| Hypotonia | 2/9 (22.22%) | 1/5 (20.00%) | 1.000 |
| Ataxia | 2/9 (22.22%) | 0/5 (0.00%) | 0.505 |
| Abnormal reflexes | 2/9 (22.22%) | 3/5 (60.00%) | 0.266 |
| Muscle weakness | 2/9 (22.22%) | 1/5 (20.00%) | 1.000 |
| Gait disturbance | 3/9 (33.33%) | 3/5 (60.00%%) | 0.580 |
| Involuntary movement | 1/9 (11.11%) | 2/5 (40.00%) | 0.505 |
| Respiratory depression | 4/9 (44.44%) | 0/5 (0.00%) | 0.221 |
| Lethargy | 1/9 (11.11%) | 0/5 (0.00%) | 1.000 |
| Dysarthria | 2/9 (22.22%) | 4/5 (80.00%) | 0.091 |
| Dysphagia | 2/9 (22.22%) | 0/5 (0.00%) | 0.505 |
| Nystagmus | 3/9 (33.33%) | 2/5 (40.00%) | 1.000 |
| Ophthalmoplegia | 4/9 (44.44%) | 3/5 (60.00%) | 1.000 |
| Ophthalmological | 5/9 (55.56%) | 3/5 (60.00%) | 1.000 |
| Gastrointestinal | 3/9 (33.33%) | 2/5 (40.00%) | 1.000 |
| Hematological | 3/9 (33.33%) | 0/5 (0.00%) | 0.258 |
| Cardiovascular | 0/9 (0.00%) | 1/5 (20.00%) | 0.357 |
| Growth | 4/9 (44.44%) | 1/5 (20.00%) | 0.580 |
| Multisystem involvement | 8/9 (88.89%) | 3/5 (60.00%) | 0.505 |
| Severe hyperlactatemia^a^ | 5/9 (55.56%) | 0/5 (0.00%) | 0.086 |
| BG | 0/9 (0.00%) | 0/5 (0.00%) | NA |
| BS | 3/9 (33.33%) | 1/5 (20.00%) | 1.000 |
| BG and BS | 6/9 (66.67%) | 4/5 (80.00%) | 1.000 |
| Medulla oblongata | 4/9 (44.44%) | 0/5 (0.00%) | 0.221 |
| Thalamus | 6/9 (66.67%) | 2/5 (40.00%) | 0.580 |
| Cerebral white matter | 2/9 (22.22%) | 1/5 (20.00%) | 1.000 |
| Cerebral cortex | 1/9 (11.11%) | 2/5 (40.00%) | 0.505 |
| Cerebral atrophy | 1/9 (11.11%) | 0/5 (0.00%) | 1.000 |
| Cerebellum | 2/9 (22.22%) | 0/5 (0.00%) | 0.505 |

**Notes:** ^a^ Severe hyperlactatemia refers to plasma lactate ≥ 3-fold upper limit of normal. All comparisons were performed using Fisher’s exact test. Asterisks indicate statistical significance without correction (^*^*p* < 0.05).

**Abbreviations:** Dev, developmental; BG, basal ganglia; BS, brainstem; NA, not applicable.

**Table S9** Results of log‑rank tests for subgroup survival analyses in the newly recruited cohort (n = 14)

| **Characteristics** | **Log-rank χ^2^** | **Uncorrected *p* value** | **FDR-corrected *p* value** |
| --- | --- | --- | --- |
| Heteroplasmy level ≥ 80% | 4.358 | 0.037 | 0.592 |
| Maternal inheritance | 1.348 | 0.246 | 0.750 |
| Age at onset ≤ 6 months | 0.340 | 0.560 | 0.759 |
| Age at onset > 2 years | 0.685 | 0.408 | 0.750 |
| Dev delay/regression | 0.558 | 0.455 | 0.750 |
| Cognitive impairment | 0.286 | 0.593 | 0.759 |
| Seizure | 1.055 | 0.304 | 0.750 |
| Ataxia | 0.451 | 0.502 | 0.759 |
| Hypertonia | 0.033 | 0.856 | 0.884 |
| Hypotonia | 10.498 | 0.0012 | 0.038^*^ |
| Myoclonus | NA | NA | NA |
| Muscle weakness | 1.704 | 0.192 | 0.750 |
| Abnormal reflexes | 0.083 | 0.774 | 0.826 |
| Gait disturbance | < 0.001 | 0.982 | 0.982 |
| Involuntary movement | 0.558 | 0.455 | 0.750 |
| Lethargy | NA | NA | NA |
| Dysarthria | 1.550 | 0.213 | 0.750 |
| Dysphagia | 0.292 | 0.589 | 0.759 |
| Nystagmus | 0.083 | 0.774 | 0.826 |
| Ophthalmoplegia | 2.242 | 0.134 | 0.750 |
| Respiratory depression | 0.642 | 0.423 | 0.750 |
| Ophthalmological | 1.550 | 0.213 | 0.750 |
| Gastrointestinal | 1.055 | 0.304 | 0.750 |
| Hematological | 0.899 | 0.343 | 0.750 |
| Cardiovascular | NA | NA | NA |
| Growth | 0.212 | 0.645 | 0.764 |
| Multisystem involvement | 0.524 | 0.469 | 0.750 |
| Severe hyperlactatemia^a^ | 0.212 | 0.645 | 0.764 |
| Brain ganglia | 0.899 | 0.343 | 0.750 |
| Brainstem | NA | NA | NA |
| Brain ganglia and brainstem | 0.899 | 0.343 | 0.750 |
| Medulla oblongata | 0.340 | 0.560 | 0.759 |
| Thalamus | 0.121 | 0.728 | 0.826 |
| Cerebral atrophy | NA | NA | NA |
| Cerebellum | 2.516 | 0.113 | 0.750 |
| Cerebral white matter | 0.558 | 0.455 | 0.750 |
| Cerebral cortex | 0.899 | 0.343 | 0.750 |

**Notes:** ^a^ Severe hyperlactatemia refers to plasma lactate ≥ 3-fold upper limit of normal. All comparisons were performed using Fisher’s exact test. Asterisks indicate statistically significant (FDR correction, ^*^*p* < 0.05).

**Abbreviations:** Dev, developmental

***Section 2 Sensitivity analysis restricted to blood‑derived heteroplasmy data (n = 33)***

**Heteroplasmy level-phenotype correlation**

Among the 33 patients with blood‑derived heteroplasmy data, the median heteroplasmy level was 73.9% (IQR 28.0%; range 14.0%–100.0%). Heteroplasmy level differed significantly across LS, LLS, and MELAS/LS groups (*p* < 0.001). Specifically, the LS group exhibited the highest heteroplasmy level (85.5% ± 10.2%), which was significantly higher than that in the LLS group (68.2% ± 15.2%; Bonferroni-corrected *p* = 0.013) and the MELAS/LS group (42.0% ± 21.0%; Bonferroni-corrected *p* < 0.001). The LLS group also had a significantly higher heteroplasmy level than the MELAS/LS group (Bonferroni-corrected *p* = 0.003) (Figure S3A). Based on clustering analysis, patients were categorized into high-, medium-, and low-level subgroups, with mean heteroplasmy levels of 88.6% ± 5.6%, 65.0% ± 8.6%, and 28.2% ± 14.2%, respectively (Figure S3B). Cross-tabulation of heteroplasmy level clusters against clinical phenotypic groups revealed cluster purities of 80.0%, 57.1%, and 75.0% for the high, medium, and low heteroplasmy level groups. Corresponding correct classification rates for LS, LLS, and MELAS/LS phenotypic groups were 80.0%, 66.7%, and 50.0%, respectively, yielding an overall correct classification rate of 69.7%. Heteroplasmy level distribution differed significantly across clinical phenotypic groups (*p* < 0.001, Fisher’s exact test). The proportion of high heteroplasmy level was significantly higher in the LS group (80.0%) than in the LLS (25.0%) and MELAS/LS groups (0%); the proportion of low heteroplasmy level was significantly higher in the MELAS/LS group (50.0%) than in the LLS (8.3%) and LS groups (0%); and the proportion of medium heteroplasmy level differed significantly between the LS and LLS groups (20.0% vs. 66.7%) (all Bonferroni‑corrected *p* < 0.05).


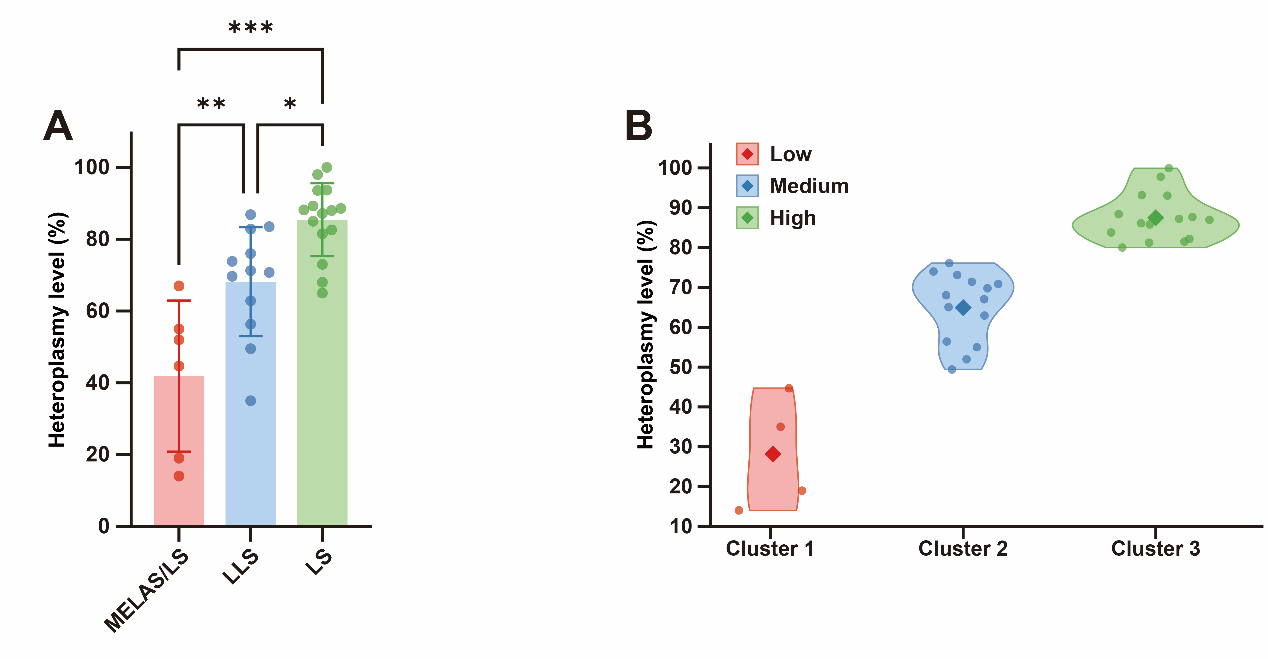


**Figure S3** Heteroplasmy-phenotype correlation restricted to blood‑derived heteroplasmy data (n = 33)

**(A)** Comparison of heteroplasmy level across clinical phenotypes (LS, LLS, MELAS/LS). Data are presented as mean ± standard deviation; Asterisks indicate statistically significant (Bonferroni correction, ^*^*p* < 0.05, ^**^*p* < 0.01, ^***^*p* < 0.001). **(B)** Clustering of patients into high-, medium-, and low-heteroplasmy level groups based on k‑means clustering. **Abbreviations:** LS, Leigh syndrome; LLS, Leigh-like syndrome; MELAS, mitochondrial encephalomyopathy with lactate acidosis and stroke-like episodes; MELAS/LS, MELAS/LS overlap syndrome.

**Disease course and prognosis**

The relevant analyses were repeated based on the blood‑derived heteroplasmy data. Bayesian logistic regression identified medulla lesions (*P* (OR > 1) = 0.94) and high heteroplasmy level (*P* (OR > 1) = 0.94) as strong risk factors for disease progression, whereas dysarthria (*P* (OR > 1) = 0.06) was a strong protective factor. Plasma lactate ≥ 3-fold ULN showed a moderate-risk tendency (*P* (OR > 1) = 0.84) (Figure S4A). Subgroup survival analysis revealed that heteroplasmy level ≥ 80% remained significantly associated with poorer survival (FDR‑corrected *p* = 0.016) (Figure S4B).


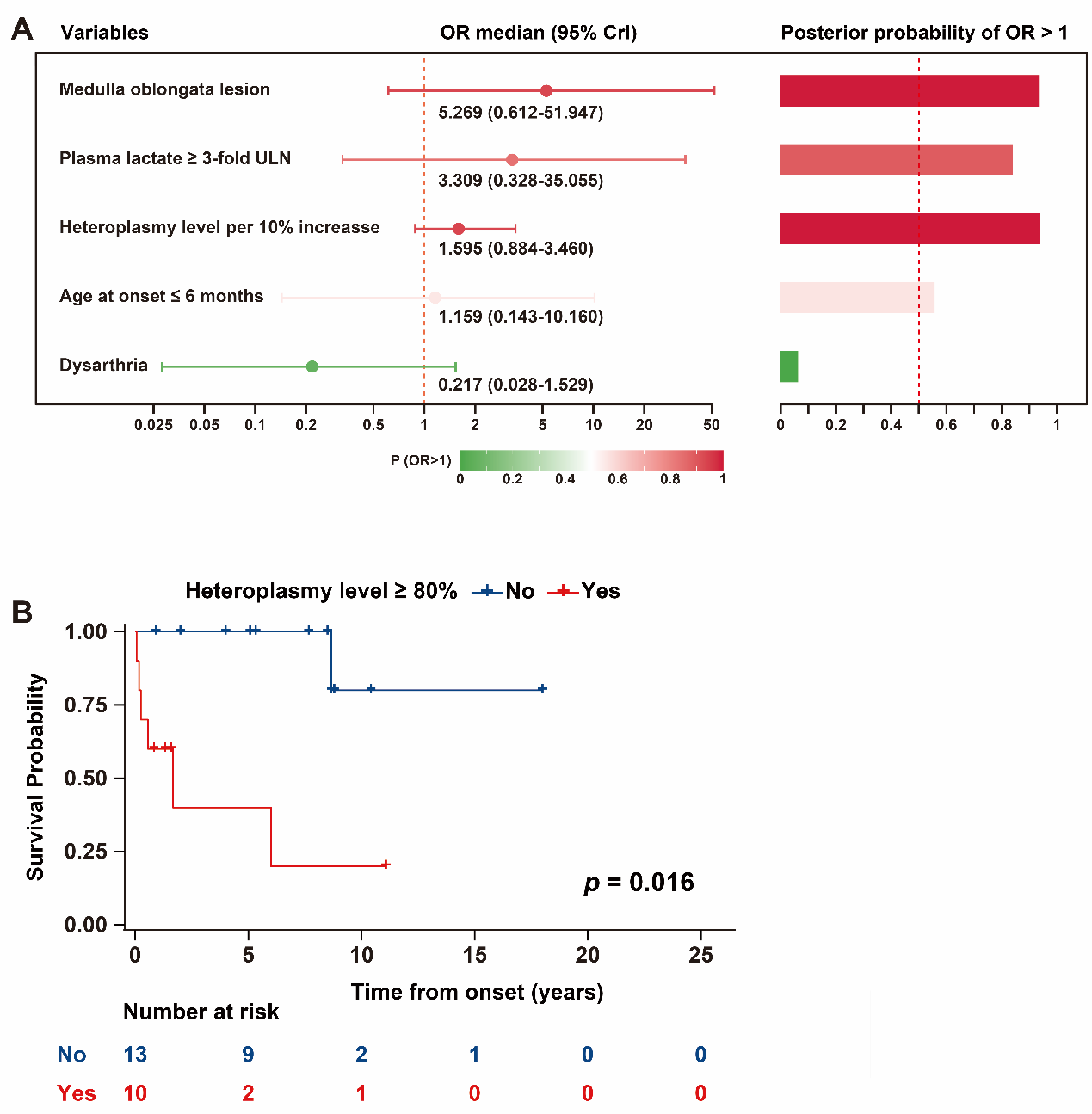


**Figure S4** Prognostic factors and survival analysis restricted to blood‑derived heteroplasmy data (n = 33)

**(A)** Multivariable Bayesian logistic regression of factors associated with disease progression. **(B)** Kaplan-Meier survival curves stratified by the presence or absence of heteroplasmy level ≥ 80%. All Log‑rank *p* values are false discovery rate‑corrected. **Abbreviations:** OR, odds ratio; CrI, credible interval; ULN, upper limit of normal.
