# Supplementary material for: Clinical Spectrum, Heteroplasmy‐Phenotype Correlation, and Prognosis of the MT‐ND3 m.10191 T > C Mutation
Source: CNS Neurosci Ther. 2026 Jun 19;32(6):e70997. doi: 10.1002/cns.70997 (PMC13280565; doi:10.1002/cns.70997)
Supplement: Supplementary file 3 — Appendix S3: Multi‐tissue comparison of heteroplasmy levels in patients with the m.10191 T > C mutation. Figure S5: Multi‐tissue comparison of heteroplasmy levels in patients with the m.10191 T > C mutation. (A) Heatmap of heteroplasmy levels across multiple tissues (blood, muscle, urine, and fibroblasts) in 13 patients with multi‐tissue data. Each column represents one patient, with patient numbers corresponding to those in the main text. Black numbers indicate patients derived from the literature, and green numbers indicate patients from the newly recruited cohort. Patients are grouped by phenotype (LS, n = 8; LLS, n = 3; MELAS/LS, n = 1; MELAS‐like, n = 1). Color intensity (blue to red) reflects heteroplasmy level (%). Missing data are indicated in gray (B–D). Comparison of heteroplasmy levels across tissue sources by phenotype. (B) LS phenotype (blood: n = 15; muscle: n = 7; fibroblasts: n = 4). One‐way ANOVA p = 0.080. (C) LLS phenotype (blood: n = 12; muscle: n = 2). (D) MELAS/LS and MELAS‐like phenotypes combined (blood: n = 6; muscle: n = 1; fibroblasts: n = 1). Data are presented as mean ± SD. Statistical comparisons were not performed for (C) and (D) due to small sample sizes. Abbreviations: LS, Leigh syndrome; LLS, Leigh‐like syndrome; MELAS, mitochondrial encephalomyopathy with lactate acidosis and stroke‐like episodes; MELAS/LS, MELAS/LS overlap syndrome. [file CNS-32-e70997-s003.docx]

**Appendix S3**

**Multi‑tissue comparison of heteroplasmy levels in patients with the m.10191T>C mutation**

***Section 1 Heteroplasmy levels across multiple tissues in the same patient***

To investigate whether heteroplasmy levels differ across tissue sources and whether such differences might affect the observed heteroplasmy‑phenotype correlation, we compared heteroplasmy levels measured in blood, muscle, fibroblasts, and urine in patients with available multi‑tissue data. A total of 13 patients had heteroplasmy data available from two or more tissue sources. These patients were classified by phenotype: LS (n = 8), LLS (n = 3), MELAS/LS (n = 1), and MELAS‑like (n = 1). A heatmap was generated to visualize the heteroplasmy levels across different tissues for each patient (Figure S5A). In the LS phenotype (n = 8), heteroplasmy levels showed no substantial differences across tissue types. In the LLS phenotype (n = 3), heteroplasmy levels in muscle and urine tended to be higher than those in blood. In the MELAS/LS and MELAS‑like phenotypes (n = 2), heteroplasmy levels in muscle were markedly higher than those in blood, with the difference being more pronounced than in the LLS group. Overall, these descriptive observations suggest that tissue‑specific differences in heteroplasmy levels may be more prominent in non‑LS phenotypes (LLS, MELAS/LS, and MELAS‑like) than in LS phenotypes. However, formal statistical comparisons were not performed due to the limited sample size and the descriptive nature of this analysis.

***Section 2 Comparison of heteroplasmy levels across tissue sources by phenotype***

In this analysis, patients were grouped by phenotype, and heteroplasmy levels from different tissue sources (blood, muscle, and fibroblasts) were compared across patients (i.e., not necessarily from the same individual). The number of patients varied by tissue source within each phenotype. Bar charts were generated for each phenotype group (Figure S5B-D). In the LS phenotype, heteroplasmy data were available from blood (n = 15), muscle (n = 7), and fibroblasts (n = 4). The mean heteroplasmy level in blood was 85.4% ± 10.2% (coefficient of variation [CV] = 11.9%, range = 65.0%–100.0%), in muscle was 86.7% ± 11.4% (CV = 13.1%, range = 69.0%–100.0%), and in fibroblasts was 70.3% ± 19.7% (CV = 28.0%, range = 50.0%–97.0%). One‑way ANOVA showed no statistically significant differences across the three tissue sources (*p* = 0.080); fibroblast‑derived heteroplasmy levels tended to be lower than those from blood and muscle, although this difference did not reach statistical significance (Figure S5B). In the LLS phenotype, heteroplasmy data were available from blood (n = 12) and muscle (n = 2), with no fibroblast data for this group. The mean heteroplasmy level in blood was 68.2% ± 15.2% (CV = 22.2%, range = 35.0%–87.0%), and in muscle was 82.5% ± 3.5% (CV = 4.3%, range = 80.0%–85.0%). Muscle‑derived heteroplasmy levels tended to be higher than those from blood, although statistical comparison was not performed due to the limited sample size in the muscle group (Figure S5C). In the MELAS/LS and MELAS‑like phenotypes (combined due to small numbers), heteroplasmy data were available from blood (n = 6), muscle (n = 1), and fibroblasts (n = 1). The mean heteroplasmy level in blood was 42.0% ± 21.0% (CV = 50.2%, range = 14.0%–67.0%), in muscle was 73.0% (n = 1), and in fibroblasts was 13.0% (n = 1). Muscle‑derived heteroplasmy was markedly higher than blood‑ and fibroblast‑derived levels, although statistical comparisons were not performed due to the limited sample size (Figure S5D). In summary, tissue‑specific differences in heteroplasmy levels were most evident in non‑LS phenotypes (LLS, MELAS/LS, and MELAS‑like), where muscle‑derived heteroplasmy tended to be higher than blood‑derived levels, whereas in the LS phenotype, heteroplasmy levels were relatively consistent across tissue sources, with fibroblasts showing a tendency toward lower values.

**
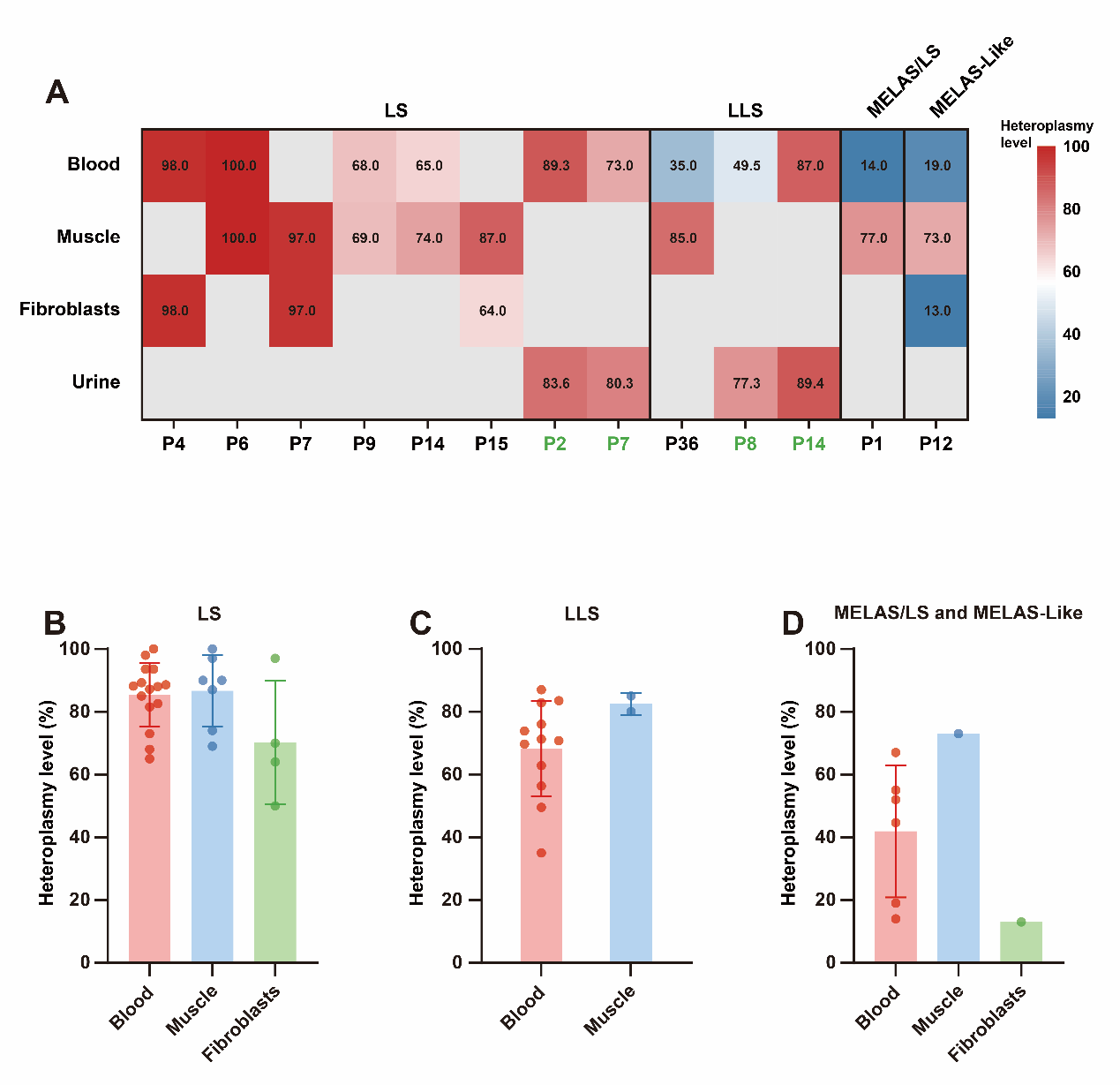
**

**Figure S5** Multi‑tissue comparison of heteroplasmy levels in patients with the m.10191T>C mutation. **(A)** Heatmap of heteroplasmy levels across multiple tissues (blood, muscle, urine, and fibroblasts) in 13 patients with multi‑tissue data. Each column represents one patient, with patient numbers corresponding to those in the main text. Black numbers indicate patients derived from the literature, and green numbers indicate patients from the newly recruited cohort. Patients are grouped by phenotype (LS, n = 8; LLS, n = 3; MELAS/LS, n = 1; MELAS‑like, n = 1). Color intensity (blue to red) reflects heteroplasmy level (%). Missing data are indicated in gray. **(B–D)** Comparison of heteroplasmy levels across tissue sources by phenotype. **(B)** LS phenotype (blood: n = 15; muscle: n = 7; fibroblasts: n = 4). One‑way ANOVA *p* = 0.080. **(C)** LLS phenotype (blood: n = 12; muscle: n = 2). **(D)** MELAS/LS and MELAS‑like phenotypes combined (blood: n = 6; muscle: n = 1; fibroblasts: n = 1). Data are presented as mean ± SD. Statistical comparisons were not performed for (C) and (D) due to small sample sizes. **Abbreviations:** LS, Leigh syndrome; LLS, Leigh-like syndrome; MELAS, mitochondrial encephalomyopathy with lactate acidosis and stroke-like episodes; MELAS/LS, MELAS/LS overlap syndrome.
