# Supplementary material for: Clinical Spectrum, Heteroplasmy‐Phenotype Correlation, and Prognosis of the MT‐ND3 m.10191 T > C Mutation
Source: CNS Neurosci Ther. 2026 Jun 19;32(6):e70997. doi: 10.1002/cns.70997 (PMC13280565; doi:10.1002/cns.70997)
Supplement: Supplementary file 4 — Table S1: Characteristics of the 38 patients with the m.10191 T > C mutation identified from the literature. Table S2: Comparison of characteristics between patients with and without cortical involvement. Table S3: Fit indices for two‐ to four‐class latent class analysis models. Table S4: Probability of each variable across the three latent classes. Table S5: Clinical characteristics of the three clusters identified by two‐step cluster analysis (n = 28). Table S6: Comparison of characteristics between patients with and without disease progression. Table S7: Results of log‐rank tests for subgroup survival analyses. [file CNS-32-e70997-s002.docx]

**Supplementary Table**

**Table S1** Characteristics of the 38 patients with the m.10191T>C mutation identified from the literature

| **No. of patient/**  **Phenotype** | **Sex/Age at onset** | **Heteroplasmy level/**  **Transmission** | **Family history** | **Clinical features** | **Brain MRI/MRS** | **Lactate** | **Muscle histology/**  **CI activity** | **Course/**  **Outcome** | **Reference** |
| --- | --- | --- | --- | --- | --- | --- | --- | --- | --- |
| 1. MELAS/LS | M/24y | 14% (B); 77% (Mu)/  Maternal | – | MA, SLE, CogI, Myoclonus, GTCS, Dystonia, Ataxia, Reflex↑, PN, OA | Midbrain, LN/U | U | N/40% (Mu) | Progression/Alive (42y) | Taylor RW et al.^[1]^ |
| 2. LS | F/Birth | 90% (Mu)/De novo | – | DD, Myoclonic jerk, Hypotonia, PS, OP, FTT, SS | Putamen/U | 8.0↑ (B);  U (CSF) | U/3.3↓ | U/U | Lebon S, et al.^[2]^ |
| 3. LLS | M/26m | 80% (Mu)/De novo | – | DD, DR, CogI, Myoclonic jerk, Hypotonia, Hypothermia, Bradypnea, Vomit, Bradycardia | GP, Putamen, Medulla, Cortico-subcortical atrophy/U | 6.0↑ (B);  U (CSF) | U/30↓ | Improvement/Alive (12y) |  |
| 4. LS | M/Birth | >98% (F, K, S, L)/ De novo | – | DD, Seizure, Hypotonia, Reflex↓, Lethargy, Hypothermia, Resp acidosis, Micrognathia, Equinovarus | Medulla, Hippocampus, Cerebellum/U | U | N/4% (F); 8% (L) | Progression/Dead (23d) | McFarland R, et al.^[3]^ |
| 5. LS | U/U | 90% (Mu); 50% (F)/ U | + | DD, CogI, and Others (Not given specifically) | BG, BS (Not given specifically) /U | Lactic acidosis | U/14% (Mu); 25% (F) | Progression/U | Bugiani M, et al.^[4]^ |
| 6. LS | M/5w | 100% (B, Mu); 50% (H)/Maternal | – | DD, Myoclonus, Hypertonia, Hypotonia, Seizure, Reflex↑, Lethargy, Bradypnea, Apnea, Resp acidosis | BG, Midbrain, Pons/U | N (B);  4.4↑ (CSF) | U/38% (Mu) | Progression/Dead (8m) | Leshinsky-Silver E, et al.^[5]^ |
| 7. LS | F/3m | 97% (Mu, F)/  Maternal | + | DD, Dysphagia, Focal seizure, GTCS, Dystonia, Hypotonia, Ataxia, Weakness, OP, FTT, GI, Resp distress | Midbrain, Thalamus/U | 5.2↑ (B);  6.1↑ (CSF) | U/↓ (Not given specifically) | Progression/Alive (22y) | Esteitie N, et al.^[6]^ |
| 8. Nonspecific MD | M/21y | 95% (Mu)/U | U | Myoclonic jerk, GTCS, EPC, Weakness, PN, OA, Cardiac arrest | Cortico-subcortical lesions, CA, Thalamus/PCr↓ | N (B);  2.4↑ (CSF) | N/23% (Mu) | Progression/Dead (27y) | Malfatti E, et al.^[7]^ |
| 9. LS | M/4m | 68% (B); 69% (Mu)/  De novo | + | DD, Seizure, Hypotonia | BG/U | Lactic acidosis | N/↓ (Not given specifically) | Progression/Dead (9y) | Bannwarth S, et al.^[8]^ |
| 10. LS | U/13m | U/U | U | DD, Myoclonus, Seizure, Spasticity, Reflex↑, GI, IM | Putamen, Thalamus, BS, CA/U | U | U/U | Stable/Alive (11y9m) | Lee HF, et al.^[9]^ |
| 11. MELAS/LS | F/5y | U/U | – | SLE, CogI, Myoclonus, Focal seizure, Dysarthria, Weakness, Ataxia, IM, HL | BG, Multifocal cortices/U | 3.2↑ (B) | N/26% (Mu) | Progression/Alive (12y) | Lim BC, et al.^[10]^ |
| 12. MELAS-like | F/U | 19% (B); 73% (Mu); 13% (F)/De novo | – | MO, SLE, CogI, Myoclonic jerk, GTCS, EPC, Ataxia, Nystagmus, FFT, SS, First-degree heart block | Peri-Rolandic and calcarine cortices/Lac↑, NAA↓ | N (B);  N (CSF) | N/N (F) | Progression/Alive (18y) | Werner KG, et al.^[11]^ |
| 13. LS | M/4m | U/U | U | Hypotonia, Nystagmus, Resp depression, Vomit, Hyperalaninemia | BG, BS (Not given specifically) /U | Lactic acidosis | Slight type I fiber prevail/↓ (Mu) | Progression/U | Lebiedzins-ka M, et al.^[12]^ |
| 14. LS | M/6m | 65% (B); 74% (Mu)/ De novo | + | DD, Seizure, Spasticity, Macrocephaly, Ataxia, Strabismus, Abnormal reflexes | Putamen, CN/U | 3.6↑ (B);  5.1↑ (CSF) | U/20% (Mu) | Stable/Alive (9y) | Nesbitt V, et al.^[13]^ |
| 15. LS | M/Birth | 87% (Mu); 64% (F); 87% (L)/De novo | – | Seizure, Hypertonia, Central hypotonia, Weakness, Lethargy, Abnormal reflexes, Apnea, Poor feeding, FFT, SS, HCM | BG, BS, Thalamus, Medulla/Lac↑ | 5.0↑ (B);  6.4↑ (CSF) | U/5% (Mu) | Progression/Dead (6w) |  |
| 16. MELAS/LS | F/11y | U/U | – | SLE, Facial spasm, GTCS, Dysphagia, Dysarthria, Dystonia, Weakness, IM, Reflex↑, Strabismus | Subcortical WM, Putamen, GP, Midbrain, Thalamus, Scattered cortices/U | 2.5↑ (B);  3.2↑ (CSF) | N/U | Progression/Alive (26y) | Matsui J, et al.^[14]^ |
| 17. LS | F/5m | >85% (B)/U | – | DD, Hypotonia, Dysphagia, Dyspnea, Pneumorrhagia, Coma | BG, CA, Cerebellum, Pons, CC/Lac↑, Cho↑, NAA↓ | 6.8↑ (B);  8.2↑ (CSF) | U/U | Progression/Dead (7m) | Danqun J, et al.^[15]^ |
| 18. LS | M/2y | U/De novo | – | DD, DR, CogI, GTCS, Dystonia, Weakness, Reflex↑, Nystagmus, Ptosis, FTT, SS | BG/U | 2.8↑ (B) | N/75% (B) | Stable/Alive (16y) | Liu YP, et al.^[16]^ |
| 19. MELAS/LS | F/11y | U/U | – | MA, SLE, Myoclonic seizure, GTCS, Abnormal reflexes, Blurred vision, HL | BG, Midbrain, Parietal and occipital cortices/U | U | N/U | Progression/Alive (19y) | Zhao D, et al.^[17]^ |
| 20. MELAS/LS | M/<1y | U/U | U | SLE, DD, DR, CogI, Seizure, Dystonia, Ataxia, OP, OA, IUGR | BG, WM, Thalamus, Cortex/Lac↑ | ↑ (B, CSF) | N/↓ (Mu) | U/U | Bannwarth S, et al.^[18]^ |
| 21. LS | M/1-16y | U/U | U | DD, CogI, Dystonia | BG, Cerebellar atrophy/U | N (B) | N/↓ (Mu, F) | U/U |  |
| 22. LS | F/4m | 70% (F)/Maternal | – | DD, DR, CogI, GTCS, Focal seizure, Dystonia, Abnormal reflexes, Poor feeding, FFT | Cortico-subcortical atrophy CA, CN, GP, Putamen, Midbrain, Thalamus/U | 5.0-9.0↑ (B) | Mitochondria↑/  0% (Mu) | Progression/Alive (25y) | Levy RJ, et al.^[19]^ |
| 23. MELAS/LS | F/18y | U/Maternal | + | SLE, Hypotonia, Focal seizure, GTCS, SE, Weakness, Reflex↓, Dysarthria | Putamen, Frontal and parietal cortices/U | 4.5↑ (B) | N/U | Stable/Alive (24y) | Li TR, et al.^[20]^ |
| 24. MELAS-like | F/42y | U/Maternal | U | SLE, Focal seizure, GTCS, Weakness, Dysarthria | Multifocal cortices, Thalamus/Lac↑, NAA↓ | U | U/U | Stable/Alive (48y) |  |
| 25. MELAS/LS | F/18y | 55% (B)/U | U | SLE, CogI, Seizure, Ataxia, PS | Multifocal cortices, WM, Putamen/U | U | U/U | U/U | Wei Y, et al.^[21]^ |
| 26. MELAS/LS | F/16y | 52% (B)/U | U | MA, SLE, Focal seizure, Ataxia, Weakness, PS, HL | Multifocal cortices, Putamen, Midbrain/ Suspicious Lac↑ | 2.8↑ (B) | U/U | Stable/U |  |
| 27. LS | M/6m | U/De novo | – | DD, Atrial septal defect, and Others (Not given specifically) | Leigh lesions (Not given specifically)/U | 7.9↑ (B) | U/U | Progression/Dead (1y) | Hu C, et al.^[22]^ |
| 28. LS | M/1.5y | 82.6% (B)/Maternal | – | DD, and Others (Not given specifically) | Leigh lesions (Not given specifically)/U | 4.6↑ (B) | U/U | Progression/U |  |
| 29. LLS | M/2m | 69.7% (B)/U | U | DD, LGS, Weakness, Multisystem involvement (GI, Endo, Renal, Resp, Ophth) | BG, BS, CA, Thalamus, Cerebellar atrophy, WM, Cortico-subcortical lesions/ Lac↑, NAA↓ | 3.3↑ (B) | N/U | U/U | Na JH, et al.^[23]^ |
| 30. LLS | F/8m | 82.9% (B)/U | U | DD, LGS, Weakness, Multisystem involvement (GI, Endo, Renal, Resp) | BG, BS, CA, Thalamus, Cortico-subcortical lesions /Lac↑ | 3.7↑ (B) | N/U | U/U |  |
| 31. LLS | F/1y | 62.9% (B)/U | U | DD, LGS, SE, Multisystem involvement (GI, Endo, Resp, Ophth) | BG, BS, CA, Thalamus, Cerebellar atrophy, WM, Cortico-subcortical lesions/ Lac↑, NAA↓ | 4.2↑ (B) | N/U | U/U |  |
| 32. LS | M/6m | 88.2% (B)/U | U | DD, Focal seizure, SE, Multisystem involvement (GI, Resp) | BG, BS, CA, Thalamus/ Lac↑ | N (B) | N/U | U/U |  |
| 33. LS | F/1y | 81.5% (B)/U | U | DD, Seizure, SE, Multisystem involvement (GI, Endo, Renal, Resp) | BG, BS/N | 3.0↑ (B) | RRF/U | U/U |  |
| 34. LS | F/3y8m | 93.6% (B)/U | U | DD, Seizure, Weakness, Multisystem involvement (GI, Resp, Ophth, HL) | BG, BS/Lac↑ | 3.1↑ (B) | N/U | U/U |  |
| 35. LLS | M/23y | 35% (B); 85% (Mu)/  Maternal | + | GTCS, Focal seizure, Spasticity, Ataxia, Weakness, Reflex↑, Dysarthria, PN, Depression, Anxiety | LN, Midbrain, Thalamus, Cortico-subcortical lesions, CA, WM/Lac↑ | U | N/U | Progression/Alive (27y) | Watson-Fargie T, et al.^[24]^ |
| 36. MELAS/LS | F/6y | 67% (B)/ De novo | U | MO, SLE, Seizure, Vomit, Impaired vision with central scotoma | BG, Multifocal cortices, Subcortical WM, Stroke-like lesions/U | 3.3↑ (B);  3.8↑ (CSF) | U/U | U/U | Borna NN, et al.^[25]^ |
| 37. LS | F/8m | 87.2% (B)/ De novo | U | DD, Poor feeding, FFT | BG, Midbrain, Thalamus, Chiari malformation/U | 6.2↑ (B);  4.6↑ (CSF) | U/U | U/U |  |
| 38. LLS | F/Birth | U/Maternal | – | MA, DD, CogI, GTCS, Focal seizure, Dystonia, Ataxia, Reflex↑, Lethargy, PN, IM, Nystagmus, Diplopia, Micropsia, Diarrhea, Resp distress, Tachycardia, PVC, Hypogonadism, Hyperlipidemia, Hypoaldosteronism, Renal insufficiency, Hemolytic anemia, HGG, Depression, Derealization, Executive dysfunction and language-finding difficulties | GP, Midbrain, WM, Face of the giant panda sign/N | Mild ↑ (B) | >10% RRF, COX-negative fibers/U | Progression/Alive (32y) | Newstead SM, et al.^[26]^ |

**Notes:** For plasma/CSF lactate, the unit is mmol/L. The reference range for normality was adopted as reported in the respective source literature. Complex I activity is presented either as the absolute enzyme activity (unit: mmol/min/mg protein) or as a percentage of the normal control value, according to the original report.

**Abbreviations:** CI, complex I; LS, Leigh syndrome; LLS, Leigh-like syndrome; MELAS, mitochondrial encephalomyopathy with lactate acidosis and stroke-like episodes; MELAS/LS, MELAS/LS overlap syndrome; MD, mitochondrial disease; M, male; F, female; d, days; w, weeks; m, months; y, years; B, blood; Mu, muscle; F, fibroblasts; K, kidney; S, spleen; L, liver; H, hair; CSF, cerebrospinal fluid; +, positive/abnormal finding; –, negative/normal finding; U, unknown/not determined; N, normal; ↑, increased; ↓, decreased; MA, migraine with aura; MO, migraine without aura; SLE, stroke-like episodes; DD, developmental delay; DR, developmental regression; CogI, cognitive impairment; GTCS, generalized tonic-clonic seizure; EPC, epilepsia partialis continua; SE, status epilepticus; LGS, Lennox-Gastaut syndrome; PN, peripheral neuropathy; IM, involuntary movement; PS, pyramidal syndrome; OA, optic atrophy; OP, ophthalmoplegia; FTT, failure to thrive; SS, short stature; IUGR, intrauterine growth failure; Resp, Respiratory; GI, gastrointestinal; HL, hearing loss; Endo, endocrine; Ophth, ophthalmology; HCM, hypertrophic cardiomyopathy; PVC, premature ventricular contraction; HGG, hypogammaglobulinemia; MRI, magnetic resonance imaging; MRS, magnetic resonance spectroscopy; BG, basal ganglia; BS, brainstem; LN, lentiform nucleus; GP, globus pallidus; CN, caudate nucleus; CC, corpus callosum; WM, white matter; CA, cerebral atrophy; PCr, phosphocreatine; Lac, lactate peak; Cho, Choline peak; NAA, N-acetylaspartic acid.

**Table S2** Comparison of characteristics between patients with and without cortical involvement

| **Characteristics** | **Without cortical involvement** | **With cortical involvement** | **Uncorrected *p* value** | **FDR-corrected *p* value** |
| --- | --- | --- | --- | --- |
| Male | 18/29 (62.07%) | 10/21 (47.62%) | 0.310^c^ | 0.568 |
| Age at onset (months) | 6 [4-18] | 72 [15-216] | < 0.001^b^ | < 0.001^***^ |
| Age at onset ≤ 6 months | 17/30 (56.67%) | 1/21 (4.76%) | < 0.001^c^ | 0.002^**^ |
| Age at onset > 2 years | 5/29 (17.24%) | 14/21 (66.67%) | < 0.001^c^ | 0.004^**^ |
| Heteroplasmy level (%) | 83.65 ± 10.05 | 58.00 ± 23.65 | < 0.001^a^ | 0.008^**^ |
| Maternal inheritance | 8/23 (34.78%) | 6/11 (54.55%) | 0.458^e^ | 0.746 |
| Dev delay/regression | 26/28 (92.86%) | 8/21 (38.10%) | < 0.001^c^ | < 0.001^***^ |
| Cognitive impairment | 12/17 (70.59%) | 8/17 (47.06%) | 0.296^e^ | 0.566 |
| Seizure | 20/28 (71.43%) | 20/21 (95.24%) | 0.079^d^ | 0.204 |
| Ataxia | 4/25 (16.00%) | 8/18 (44.44%) | 0.040^c^ | 0.161 |
| Myoclonus | 3/25 (12.00%) | 6/17 (35.29%) | 0.155^d^ | 0.341 |
| Hypertonia | 14/25 (56.00%) | 7/18 (38.89%) | 0.268^c^ | 0.536 |
| Hypotonia | 11/25 (44.00%) | 2/18 (11.11%) | 0.021^c^ | 0.129 |
| Abnormal muscle tone | 20/25 (80.00%) | 9/18 (50.00%) | 0.038^c^ | 0.161 |
| Abnormal reflexes | 12/25 (48.00%) | 9/18 (50.00%) | 0.897^c^ | 1.000 |
| Muscle weakness | 6/28 (21.43%) | 10/21 (47.62%) | 0.053^c^ | 0.161 |
| Gait disturbance | 6/15 (40.00%) | 2/15 (13.33%) | 0.215^e^ | 0.450 |
| Involuntary movement | 3/25 (12.00%) | 4/18 (22.22%) | 0.633^d^ | 0.871 |
| Lethargy | 5/25 (20.00%) | 0/18 (0.00%) | 0.124^d^ | 0.288 |
| Brainstem dysfunction^f^ | 20/26 (76.92%) | 14/18 (77.78%) | 1.000^d^ | 1.000 |
| Dysarthria | 5/25 (20.00%) | 6/18 (33.33%) | 0.526^d^ | 0.805 |
| Dysphagia | 4/25 (16.00%) | 1/18 (5.56%) | 0.567^d^ | 0.805 |
| Respiratory depression | 10/25 (40.00%) | 2/18 (11.11%) | 0.037^c^ | 0.161 |
| Nystagmus | 7/27 (25.93%) | 2/19 (10.53%) | 0.358^d^ | 0.613 |
| Ophthalmoplegia | 9/27 (33.33%) | 4/19 (21.05%) | 0.362^c^ | 0.613 |
| Ophthalmological | 13/28 (46.43%) | 11/21 (52.38%) | 0.680^c^ | 0.896 |
| Gastrointestinal | 14/28 (50.00%) | 5/21 (23.81%) | 0.063^c^ | 0.712 |
| Hematological | 4/25 (16.00%) | 1/18 (5.56%) | 0.567^d^ | 0.805 |
| Cardiovascular | 4/29 (13.79%) | 3/21 (14.29%) | 1.000^d^ | 1.000 |
| Genitourinary | 3/28 (10.71%) | 2/21 (9.52%) | 1.000^d^ | 1.000 |
| Endocrine | 3/28 (10.71%) | 3/21 (14.29%) | 1.000^d^ | 1.000 |
| Growth | 11/25 (44.00%) | 2/18 (11.11%) | 0.021^c^ | 0.129 |
| Auditory | 2/28 (7.14%) | 3/21 (14.29%) | 0.733^d^ | 0.896 |
| Multisystem involvement | 25/28 (89.29%) | 13/21 (61.90%) | 0.054^d^ | 0.161 |
| Severe hyperlactatemia^g^ | 12/29 (41.38%) | 1/15 (6.67%) | 0.041^d^ | 0.161 |
| Prognosis | 19/25 (76.00%) | 10/15 (66.67%) | 0.784^d^ | 0.932 |
| BG | 23/29 (79.31%) | 17/21 (80.95%) | 1.000^d^ | 1.000 |
| BS | 24/29 (82.76%) | 13/21 (61.90%) | 0.097^c^ | 0.237 |
| BG and BS | 18/29 (62.07%) | 12/21 (57.14%) | 0.726^c^ | 0.896 |
| Medulla oblongata | 5/29 (17.24%) | 2/21 (9.52%) | 0.716^d^ | 0.896 |
| Thalamus | 10/29 (34.48%) | 13/21 (61.90%) | 0.055^c^ | 0.161 |
| Cerebral atrophy | 5/29 (17.24%) | 6/21 (28.57%) | 0.543^d^ | 0.805 |
| Cerebellum | 4/29 (13.79%) | 3/21 (14.29%) | 1.000^d^ | 1.000 |
| Cerebral white matter | 3/29 (10.34%) | 8/21 (38.10%) | 0.046^d^ | 0.161 |

**Notes:** ^a^ *p* value was calculated using independent t-test. ^b^ *p* value was calculated using Mann-Whitney U test. ^c^ *p* value was calculated using Chi-squared test. ^d^ *p* value was calculated using Corrected chi-square test. ^e^ *p* value was calculated using Fisher exact test. ^f^ Brainstem dysfunction encompassed respiratory depression, nystagmus, ophthalmoplegia, dysphagia, and dysarthria. ^g^ Severe hyperlactatemia refers to plasma lactate ≥ 3-fold upper limit of normal. Asterisks indicate statistically significant (FDR correction, ^*^*p* < 0.05, ^**^*p* < 0.01, ^***^*p* < 0.001).

**Abbreviations:** FDR, false discovery rate correction; Dev, developmental; BG, basal ganglia; BS, brainstem.

**Table S3** Fit indices for two‑ to four‑class latent class analysis models

| **Model statistics** | **Two-class solution** | **Three-class solution** | **Four-class solution** |
| --- | --- | --- | --- |
| AIC | 581.022 | 568.330 | 580.416 |
| BIC | 637.029 | 653.243 | 694.236 |
| Adjusted BIC | 539.857 | 505.919 | 496.758 |
| Entropy | 1.000 | 1.000 | 1.000 |
| Log-likelihood | -259.511 | -237.165 | -227.208 |
| LMR-LRT (*p* value) | < 0.001 | 0.050 | 0.108 |
| BLRT (*p* value) | < 0.001 | < 0.001 | 0.500 |

**Abbreviations:** AIC, Akaike information criterion; BIC, Bayesian information criterion; LMR-LRT, Lo-Mendell-Rubin Likelihood Ratio Test; BLRT, Bootstrap Likelihood Ratio Test.

**Table S4** Probability of each variable across the three latent classes

| **Variable** | **Class 1** | **Class 2** | **Class 3** |
| --- | --- | --- | --- |
| Phenotype |  |  |  |
| LS | 0.000 | 0.000 | 1.000 |
| LLS | 0.131 | 0.917 | 0.000 |
| MELAS/LS | 0.869 | 0.083 | 0.000 |
| Heteroplasmy level |  |  |  |
| Low-level | 0.754 | 0.222 | 0.000 |
| Medium-level | 0.246 | 0.444 | 0.187 |
| High-level | 0.000 | 0.334 | 0.812 |
| Ataxia | 0.462 | 0.300 | 0.062 |
| Hypotonia | 0.154 | 0.100 | 0.562 |
| Growth disorder | 0.231 | 0.200 | 0.500 |
| Age of onset > 2 years | 1.000 | 0.091 | 0.105 |
| Respiratory depression | 0.000 | 0.417 | 0.353 |
| Plasma lactate ≥ 3-fold ULN | 0.000 | 0.273 | 0.556 |
| Developmental delay/regression | 0.000 | 1.000 | 1.000 |
| Cerebral cortex lesion | 0.869 | 0.583 | 0.000 |
| Cerebral white matter lesion | 0.308 | 0.583 | 0.167 |

**Abbreviations:** LS, Leigh syndrome; LLS, Leigh-like syndrome; MELAS, mitochondrial encephalomyopathy with lactate acidosis and stroke-like episodes; MELAS/LS, MELAS/LS overlap syndrome; ULN, upper limit of normal.

**Table S5** Clinical characteristics of the three clusters identified by two‑step cluster analysis (n = 28)

| **Variable** | **Group 1**  **(n = 6)** | **Group 2**  **(n = 12)** | **Group 3**  **(n = 10)** | ***p* value** |
| --- | --- | --- | --- | --- |
| Phenotype | | | | < 0.001^***^ |
| LS | 0 (0.00%) | 5 (41.67%) | 10 (100.00%) |  |
| LLS | 2 (33.33%) | 7 (58.33%) | 0 (0.00%) |  |
| MELAS/LS | 4 (66.67%) | 0 (0.00%) | 0 (0.00%) |  |
| Heteroplasmy level | | | | 0.017^*^ |
| Low-level | 3 (50.00%) | 3 (25.00%) | 0 (0.00%) |  |
| Medium-level | 3 (50.00%) | 4 (33.33%) | 2 (20.00%) |  |
| High-level | 0 (0.00%) | 5 (41.67%) | 8 (80.00%) |  |
| Ataxia | 3 (50.00%) | 4 (33.33%) | 1 (10.00%) | 0.207 |
| Hypotonia | 1 (16.67%) | 1 (8.33%) | 7 (70.00%) | 0.007^**^ |
| Growth disorder | 3 (50.00%) | 1 (8.33%) | 6 (60.00%) | 0.031^*^ |
| Age at onset > 2 years | 6 (100.00%) | 2 (16.67%) | 0 (0.00%) | < 0.001^***^ |
| Respiratory depression | 0 (0.00%) | 3 (25.00%) | 5 (50.00%) | 0.145 |
| Plasma lactate ≥ 3-fold ULN | 0 (0.00%) | 4 (33.33%) | 7 (70.00%) | 0.017^*^ |
| Developmental delay/regression | 0 (0.00%) | 12 (100.00%) | 10 (100.00%) | < 0.001^***^ |
| Cerebral cortical lesions | 4 (66.67%) | 6 (50.00%) | 0 (0.00%) | 0.004^**^ |
| Cerebral white matter lesions | 1 (16.67%) | 3 (25.00%) | 0 (0.00%) | 0.238 |

**Notes:** All comparisons were performed using Fisher’s exact test. Asterisks indicate statistical significance without correction (^*^*p* < 0.05, ^**^*p* < 0.01, ^***^*p* < 0.001).

**Abbreviations:** LS, Leigh syndrome; LLS, Leigh-like syndrome; MELAS, mitochondrial encephalomyopathy with lactate acidosis and stroke-like episodes; MELAS/LS, MELAS/LS overlap syndrome; ULN, upper limit of normal.

**Table S6** Comparison of characteristics between patients with and without disease progression

| **Characteristics** | **Progression** | **Non-progression** | **Uncorrected *p* value** |
| --- | --- | --- | --- |
| Male | 17/26 (65.38%) | 5/10 (50.00%) | 0.462 |
| Onset age < 6m | 13/26 (50.00%) | 1/11 (9.09%) | 0.027^*^ |
| Onset age > 2y | 10/26 (38.46%) | 6/11 (54.55%) | 0.475 |
| Heteroplasmy level ≥ 80% | 13/21 (61.90%) | 0/7 (0.00%) | 0.007^**^ |
| Maternal | 9/20 (45.00%) | 4/9 (44.44%) | 1.000 |
| Migraine | 4/26 (15.38%) | 2/11 (18.18%) | 1.000 |
| Stroke-like episodes | 5/26 (19.23%) | 4/11 (36.36%) | 0.404 |
| Dev delay/regression | 15/23 (65.22%) | 6/11 (54.55%) | 0.709 |
| Cognitive impairment | 12/17 (70.59%) | 4/11 (36.36%) | 0.121 |
| Seizure | 22/26 (84.62%) | 8/11 (72.73%) | 0.403 |
| Myoclonus | 7/26 (26.92%) | 1/10 (10.00%) | 0.397 |
| Hypertonia | 13/26 (50.00%) | 6/11 (54.55%) | 1.000 |
| Hypotonia | 10/26 (38.46%) | 2/11 (18.18%) | 0.279 |
| Ataxia | 8/26 (30.77%) | 2/11 (18.18%) | 0.688 |
| Abnormal reflexes | 11/26 (42.31%) | 8/11 (72.73%) | 0.151 |
| Muscle weakness | 8/26 (30.77%) | 5/11 (45.45%) | 0.465 |
| Gait disturbance | 4/18 (22.22%) | 4/9 (44.44%) | 0.375 |
| Involuntary movement | 4/26 (15.38%) | 3/11 (27.27%) | 0.403 |
| Respiratory depression | 12/26 (46.15%) | 0/11 (0.00%) | 0.007^**^ |
| Lethargy | 5/26 (19.23%) | 0/11 (0.00%) | 0.295 |
| Dysarthria | 5/26 (19.23%) | 6/11 (54.55%) | 0.051 |
| Dysphagia | 5/26 (19.23%) | 0/11 (0.00%) | 0.295 |
| Nystagmus | 6/26 (23.08%) | 3/11 (27.27%) | 1.000 |
| Ophthalmoplegia | 7/26 (26.92%) | 4/11 (36.36%) | 0.699 |
| Ophthalmological | 13/26 (50.00%) | 5/11 (45.45%) | 1.000 |
| Gastrointestinal | 9/26 (34.62%) | 3/11 (27.27%) | 1.000 |
| Hematological | 5/26 (19.23%) | 0/11 (0.00%) | 0.295 |
| Cardiovascular | 5/26 (19.23%) | 1/11 (9.09%) | 0.646 |
| Growth | 8/26 (30.77%) | 2/11 (18.18%) | 0.688 |
| Multisystem involvement | 22/26 (84.62%) | 7/11 (63.64%) | 0.203 |
| Severe hyperlactatemia^a^ | 9/22 (40.91%) | 0/9 (0.00%) | 0.032^*^ |
| BG | 2/26 (7.69%) | 3/11 (27.27%) | 0.144 |
| BS | 5/26 (19.23%) | 2/11 (18.18%) | 1.000 |
| BG and BS | 17/26 (65.38%) | 5/11 (45.45%) | 0.295 |
| Medulla oblongata | 7/26 (26.92%) | 0/11 (0.00%) | 0.080 |
| Thalamus | 12/26 (46.15%) | 5/11 (45.45%) | 1.000 |
| Cerebral white matter | 5/26 (19.23%) | 1/11 (9.09%) | 0.646 |
| Cerebral cortex | 8/26 (30.77%) | 5/11 (45.45%) | 0.465 |
| Cerebral atrophy | 6/26 (23.08%) | 1/11 (9.09%) | 0.649 |
| Cerebellum | 4/26 (15.38%) | 0/11 (0.00%) | 0.296 |

**Notes:** ^a^ Severe hyperlactatemia refers to plasma lactate ≥ 3-fold upper limit of normal. All comparisons were performed using Fisher’s exact test. Asterisks indicate statistical significance without correction (^*^*p* < 0.05, ^**^*p* < 0.01).

**Abbreviations:** Dev, developmental; BG, basal ganglia; BS, brainstem.

**Table S7** Results of log‑rank tests for subgroup survival analyses

| **Characteristics** | **Log-rank χ^2^** | **Uncorrected *p* value** | **FDR-corrected *p* value** |
| --- | --- | --- | --- |
| Heteroplasmy level ≥ 80% | 9.160 | 0.002 | 0.030^*^ |
| Maternal inheritance | 2.866 | 0.090 | 0.335 |
| Age at onset ≤ 6 months | 7.408 | 0.006 | 0.060 |
| Age at onset > 2 years | 4.482 | 0.034 | 0.181 |
| Dev delay/regression | 1.571 | 0.210 | 0.432 |
| Cognitive impairment | 0.081 | 0.776 | 0.969 |
| Seizure | 0.011 | 0.915 | 0.969 |
| Ataxia | 3.143 | 0.076 | 0.313 |
| Hypertonia | 1.347 | 0.246 | 0.455 |
| Hypotonia | 19.143 | < 0.001 | < 0.001^***^ |
| Myoclonus | 0.010 | 0.919 | 0.969 |
| Muscle weakness | 0.118 | 0.731 | 0.969 |
| Abnormal reflexes | 0.029 | 0.866 | 0.969 |
| Gait disturbance | 0.014 | 0.907 | 0.969 |
| Involuntary movement | 2.355 | 0.125 | 0.385 |
| Lethargy | 5.630 | 0.018 | 0.109 |
| Dysarthria | 3.860 | 0.049 | 0.229 |
| Dysphagia | 0.003 | 0.960 | 0.969 |
| Nystagmus | 0.714 | 0.398 | 0.701 |
| Ophthalmoplegia | 0.086 | 0.769 | 0.969 |
| Respiratory depression | 6.207 | 0.013 | 0.094 |
| Ophthalmological | 0.544 | 0.461 | 0.775 |
| Gastrointestinal | 1.600 | 0.206 | 0.432 |
| Hematological | 1.682 | 0.195 | 0.432 |
| Cardiovascular | 1.419 | 0.234 | 0.455 |
| Growth | 0.060 | 0.807 | 0.969 |
| Multisystem involvement | 0.002 | 0.969 | 0.969 |
| Severe hyperlactatemia^a^ | 1.614 | 0.204 | 0.432 |
| Brain ganglia | 0.022 | 0.881 | 0.969 |
| Brainstem | 0.003 | 0.955 | 0.969 |
| Brain ganglia and brainstem | 0.169 | 0.681 | 0.969 |
| Medulla oblongata | 2.103 | 0.147 | 0.388 |
| Thalamus | 0.312 | 0.576 | 0.927 |
| Cerebral atrophy | 0.019 | 0.889 | 0.969 |
| Cerebellum | 14.345 | < 0.001 | 0.003^**^ |
| Cerebral white matter | 2.117 | 0.146 | 0.388 |
| Cerebral cortex | 2.522 | 0.112 | 0.378 |

**Notes:** ^a^ Severe hyperlactatemia refers to plasma lactate ≥ 3-fold upper limit of normal. All comparisons were performed using Fisher’s exact test. Asterisks indicate statistically significant (FDR correction, ^*^*p* < 0.05, ^**^*p* < 0.01, ^***^*p* < 0.001).

**Abbreviations:** Dev, developmental

**References**

[1] Taylor R W, Singh-Kler R, Hayes C M, et al. Progressive mitochondrial disease resulting from a novel missense mutation in the mitochondrial DNA ND3 gene [J]. Annals of neurology, 2001, 50(1): 104-7.

[2] Lebon S, Chol M, Benit P, et al. Recurrent de novo mitochondrial DNA mutations in respiratory chain deficiency [J]. Journal of medical genetics, 2003, 40(12): 896-9.

[3] McFarland R, Kirby D M, Fowler K J, et al. De novo mutations in the mitochondrial ND3 gene as a cause of infantile mitochondrial encephalopathy and complex I deficiency [J]. Annals of neurology, 2004, 55(1): 58-64.

[4] Bugiani M, Invernizzi F, Alberio S, et al. Clinical and molecular findings in children with complex I deficiency [J]. Biochimica et biophysica acta, 2004, 1659(2-3): 136-47.

[5] Leshinsky-Silver E, Lev D, Tzofi-Berman Z, et al. Fulminant neurological deterioration in a neonate with Leigh syndrome due to a maternally transmitted missense mutation in the mitochondrial ND3 gene [J]. Biochemical and Biophysical Research Communications, 2005, 334(2): 582-7.

[6] Esteitie N, Hinttala R, Wibom R, et al. Secondary metabolic effects in complex I deficiency [J]. Annals of neurology, 2005, 58(4): 544-52.

[7] Malfatti E, Bugiani M, Invernizzi F, et al. Novel mutations of ND genes in complex I deficiency associated with mitochondrial encephalopathy [J]. Brain : a journal of neurology, 2007, 130(Pt 7): 1894-904.

[8] Bannwarth S, Procaccio V, Rouzier C, et al. Rapid identification of mitochondrial DNA (mtDNA) mutations in neuromuscular disorders by using surveyor strategy [J]. Mitochondrion, 2008, 8(2): 136-45.

[9] Lee H F, Tsai C R, Chi C S, et al. Leigh syndrome: clinical and neuroimaging follow-up [J]. Pediatric neurology, 2009, 40(2): 88-93.

[10] Lim B C, Park J D, Hwang H, et al. Mutations in ND subunits of complex I are an important genetic cause of childhood mitochondrial encephalopathies [J]. Journal of child neurology, 2009, 24(7): 828-32.

[11] Werner K G, Morel C F, Kirton A, et al. Rolandic mitochondrial encephalomyelopathy and MT-ND3 mutations [J]. Pediatric neurology, 2009, 41(1): 27-33.

[12] Lebiedzinska M, Karkucinska-Wieckowska A, Giorgi C, et al. Oxidative stress-dependent p66Shc phosphorylation in skin fibroblasts of children with mitochondrial disorders [J]. Biochimica et Biophysica Acta (BBA) - Bioenergetics, 2010, 1797(6): 952-60.

[13] Nesbitt V, Morrison P J, Crushell E, et al. The clinical spectrum of the m.10191T>C mutation in complex I-deficient Leigh syndrome [J]. Developmental medicine and child neurology, 2012, 54(6): 500-6.

[14] Matsui J, Takano T, Ryujin F, et al. [A case of mitochondrial myopathy, encephalopathy, lactic acidosis and stroke-like episode/Leigh overlap syndrome] [J]. No to hattatsu = Brain and development, 2014, 46(5): 363-6.

[15] Danqun J, Jie D, Wenjia T, et al. [Clinical characteristics and genetic analysis of two cases with Leigh syndrome with acute pulmonary hemorrhage as predominant manifestation] [J]. Zhonghua er ke za zhi = Chinese journal of pediatrics, 2015, 53(4): 290-5.

[16] Liu Y P, Ma Y Y, Wu T F, et al. [Mitochondrial respiratory chain complex I deficiency due to 10191T>C mutation in ND3 gene] [J]. Zhongguo dang dai er ke za zhi = Chinese journal of contemporary pediatrics, 2012, 14(8): 561-6.

[17] Zhao D, Hong D, Zhang W, et al. Mutations in mitochondrially encoded complex I enzyme as the second common cause in a cohort of Chinese patients with mitochondrial myopathy, encephalopathy, lactic acidosis and stroke-like episodes [J]. Journal of human genetics, 2011, 56(11): 759-64.

[18] Bannwarth S, Procaccio V, Lebre A S, et al. Prevalence of rare mitochondrial DNA mutations in mitochondrial disorders [J]. Journal of medical genetics, 2013, 50(10): 704-14.

[19] Levy R J, Ríos P G, Akman H O, et al. Long survival in patients with leigh syndrome and the m.10191T>C mutation in MT-ND3 : a case report and review of the literature [J]. Journal of child neurology, 2014, 29(10): Np105-10.

[20] Li T R, Wang Q, Liu M M, et al. A Chinese Family With Adult-Onset Leigh-Like Syndrome Caused by the Heteroplasmic m.10191T>C Mutation in the Mitochondrial MTND3 Gene [J]. Frontiers in neurology, 2019, 10: 347.

[21] Wei Y, Cui L, Peng B. Mitochondrial DNA mutations in late-onset Leigh syndrome [J]. Journal of neurology, 2018, 265(10): 2388-95.

[22] Hu C, Li X, Zhao L, et al. Clinical and molecular characterization of pediatric mitochondrial disorders in south of China [J]. European journal of medical genetics, 2020, 63(8): 103898.

[23] Na J H, Lee M J, Lee C H, et al. Association Between Epilepsy and Leigh Syndrome With MT-ND3 Mutation, Particularly the m.10191T>C Point Mutation [J]. Frontiers in neurology, 2021, 12: 752467.

[24] Watson-Fargie T, Marshall V, Fullerton N E, et al. Leigh syndrome: an adult presentation of a paediatric disease [J]. Practical neurology, 2024, 24(1): 45-50.

[25] Borna N N, Kishita Y, Shimura M, et al. Identification of a novel MT-ND3 variant and restoring mitochondrial function by allotopic expression of MT-ND3 gene [J]. Mitochondrion, 2024, 76: 101858.

[26] Newstead S M, Finsterer J. Leigh-Like Syndrome With a Novel, Complex Phenotype Due to m.10191T>C in Mt-ND3 [J]. Cureus, 2022, 14(9): e28986.
